# Impact of soil moisture content on urban tree evaporative cooling and human thermal comfort

**DOI:** 10.1038/s42949-025-00220-0

**Published:** 2025-05-16

**Authors:** L. Gobatti, P. M. Bach, M. Maurer, J. P. Leitão

**Affiliations:** 1 Swiss Federal Institute of Aquatic Science & Technology (Eawag), Dübendorf, ZH Switzerland; 2 Institute of Environmental Engineering, ETH Zürich, Zurich, ZH Switzerland; 3 Department of Civil Engineering, Monash University, Clayton, VIC Australia; 4 EdenCT, Dübendorf, ZH Switzerland

**Keywords:** Environmental sciences, Engineering

## Abstract

Urban temperatures are rising, and urban trees can help mitigate the consequences of heat stress. However, the influence of water availability on the evaporative cooling efficiency of trees across diverse urban settings remains insufficiently understood. We modelled how varying soil moisture, built environment and tree amounts affect human thermal comfort. Our results show that increasing tree cover and maintaining high soil moisture through irrigation can generate areas of ‘no thermal stress’ in Zurich during an average summer day, primarily via direct soil evaporation and in less dense Local Climate Zones. In denser built environments and without enough soil moisture, achieving such thermal comfort proved more challenging. On extreme summer days, however, even extensive tree planting and full irrigation were insufficient to alleviate heat stress, indicating the need for additional adaptation strategies. Our study underscores the critical but limited role of tree planting and water management in mitigating urban heat, offering practical recommendations for green infrastructure managers.

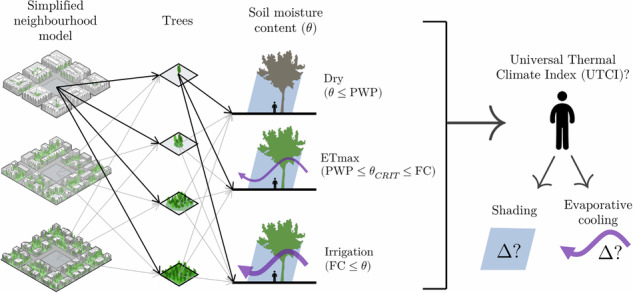

## Introduction

Urbanisation leads to land cover and land use changes that increase local heat absorption and heat trapping, as buildings are often made of materials with higher thermal capacity and lower albedo compared to vegetated areas^1^. The smaller amounts of vegetated surfaces also contribute to the reduction of the overall volume evapotranspiration in urban areas^2^, while the presence of buildings increases the overall landscape surface roughness, reducing air circulation. These factors contribute to the Urban Heat Island (UHI) effect^3^, which describes the systematically higher urban temperatures in comparison with their rural surroundings^4^. UHI effects are even being further amplified by the increasing frequency and intensity of heatwaves globally^5^.

Long heat exposure directly affects the quality of life of urban citizens, increasing health risks, straining healthcare systems and significantly increasing heat-related mortality rates^6,7^. In addition, there is a statistically significant link between UHI intensity and neighbourhoods characterised by lower socioeconomic status^2^, which are likewise areas with lower provision of passive cooling services, such as urban greenery^6,8^. These inequalities lead to fundamental spatial environmental injustice and higher heat-related morbidity and mortality in these areas^9^. In absence of passive cooling solutions, citizens resort to active cooling solutions, such as air-conditioning, as a coping mechanism to heat waves^10^. The use of active cooling increases anthropogenic heat generation, thereby further deteriorating outdoor thermal comfort^11^. Mitigating heat hazards in cities becomes, thus, a complex public health issue that encompasses physiological factors along with social, ecological, and urban spatial variables^12^; this highlights the need to explore the potential of urban greenery as a passive cooling solution.

Towards that direction, a considerable amount of research supports the use of Blue-Green Infrastructure (BGI) to mitigate urban heat^13–15^. BGI combines the use of vegetation (green) and water features (blue) to create a synergistic effect that enhances passive urban cooling^16^. Other terminologies such as Nature-based Solutions, Green Infrastructure and Urban Greenery can also refer to similar climate mitigation and adaptation structures, depending on disciplinary and geographical contexts^17^. Research indicates that BGI can lower ambient temperatures by regulating surface energy exchange processes, influencing air movement and heat dispersion, while also providing multifunctional ecosystem services^13,18^.

The main mechanisms of BGI to mitigate heat are shading and evaporative cooling (EC)^19^. Shading refers to the interception of short-wave solar radiation by vegetation canopy, which reduces heat absorption, heat storage, and the emission of long-wave radiation from shaded built surfaces, while providing substantial reduction of mean radiant temperatures^20^. EC results from evapotranspiration (ET), sometimes referred to as adiabatic cooling, which involves not only the transfer of water from plants to the atmosphere (biotic flux), but also from soil to atmosphere (abiotic flux). ET combines evaporation from plant canopy transpiration (${E}_{T}$), evaporation from plant canopy interception (${E}_{I}$) and direct soil surface evaporation (${E}_{S}$)^21,22^.

**Fig. 1:**
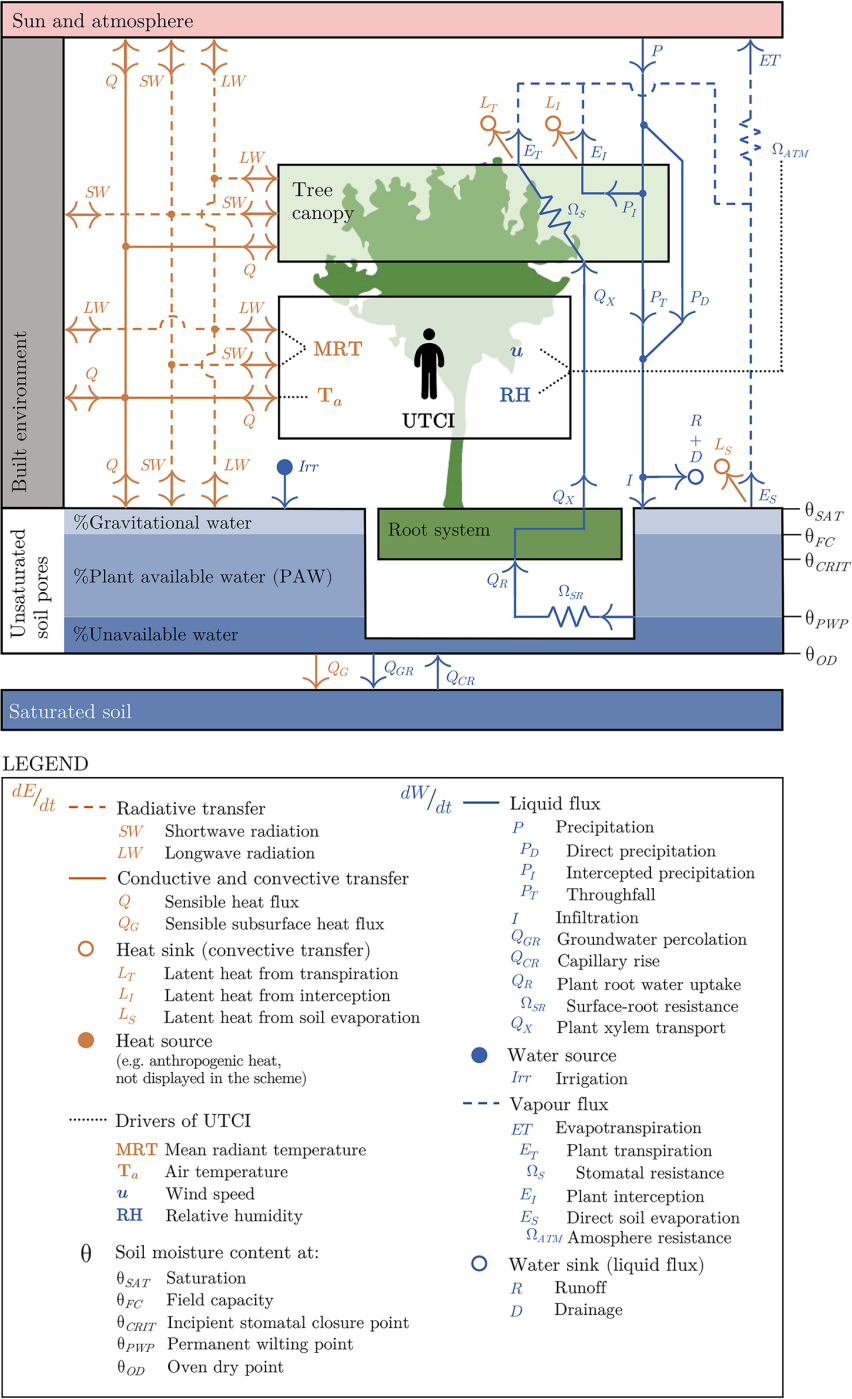
Main fluxes in the energy (${\rm{dE}}/{\rm{dt}}$) and water (${\rm{dW}}/{\rm{dt}}$) balance of the vegetation-surface-atmosphere continuum and their influences on UTCI. Direct and diffuse shortwave radiation is not decoupled for simplification. Based on Rodríguez-Iturbe and Porporato^30^; Schymanski et al.^77^; Seneviratne et al.^31^, and Verstraeten et al., 2008^30,31,77,88^.

Having a direct link with ET, the global water cycle and the global energy cycles are closely coupled. Water significantly influences the global energy balance^23^: more than 50% of the solar radiation absorbed by land surfaces is used in EC processes^20^. In the global water balance, the total volume of water moved by biotic and abiotic ET fluxes represents more than 60% of the total precipitation on Earth^20,24^. At the human scale, the heat transfer processes between our bodies and their outdoor surroundings involve complex interactions of conduction, convection, radiation, and evaporation^25^. To translate these processes into a subjective thermal comfort index, they need to be integrated into an 'equivalent temperature'^26^. These indices allow individuals to compare the combined effects of outdoor heat transfer processes to an equivalent indoor temperature^26^. One of these indexes is the Universal Thermal Climate Index (UTCI), which considers factors such as clothing insulation, evaporative resistance, and body coverage^27^. In comparison to other indicators, such as the Physiological Equivalent Temperature (PET), UTCI is more sensitive to relative humidity, which plays an important role in assessing EC^28^. Figure 1 presents the schematic integration of the heat and water cycles for a microclimatic urban setting we are proposing in this study.

The coupling of water and heat in plant transpiration (${E}_{T}$) happens via the conversion of liquid water into water vapour through vegetation stomata, which transforms sensible into latent heat, decreasing leaf and surrounding air temperatures^19,26^. The same process of sinking heat via conversion of sensible to latent heat happens for plant interception (${E}_{I}$) and direct soil evaporation (${E}_{S}$). This cooler air can be advected to surrounding areas, producing a cooling effect attributable to the BGI^20^. Physical attributes of tree species, including tree shape, canopy size, canopy density, and leaf characteristics^29^, as well as soil characteristics, including soil matric potential and water availability, affect the resulting cooling effect^30^.

The EC processes can be either water-limited or energy-limited^31^. For the case of ${E}_{S}$, when soils are unsaturated, which is typically the case of superficial layers of soil in natural environments, evaporation from bare soil surfaces is more heavily affected by soil moisture content and subsoil physical properties than by atmospheric demand^32^. Soil moisture content, thus, is a critical factor in ET, as sufficient moisture ensures continuous water availability for ${E}_{T}$ and ${E}_{S}$, enhancing the cooling effect^21,33^. Ventilation, which is directly influenced by local topography, climate, and the surrounding built environment’s morphology, is also crucial for vegetation, as it removes moisture-laden still air, allowing for continued EC and efficient water vapour diffusion from the leaves into the surrounding air^19^. Urban vegetation ET is therefore highly influenced by its immediate urban surrounding conditions.

For extreme summer weather conditions, such as heatwave days, many cities are building cooling centres as well as temporarily adapting public places to shelter the population experiencing heat vulnerability by using active cooling^34,35^. To evaluate passive cooling options, such as urban greenery, better understanding of plant cooling performance under heatwave days is required^36^. In this scenario, investigating minimum irrigation upkeep could offer valuable insights to plant selection and maintenance regimes, as not all species can provide the same cooling performance under extreme temperatures^14^.

The EC attributable to urban plants involves complex interactions between soil, water, atmosphere, vegetation and built environment, requiring comprehensive approaches that parameterise the most relevant variables driving vegetation’s effect on urban microclimate^37^. Recent studies analysed the combination of some of those interactions, such as between vegetation cooling and urbanisation intensity^38^, atmospheric conditions, vegetation amount^39^ and vegetation configuration^40–42^. However, taking all those interactions into account in a single study, with the same control scenarios, is yet a gap to be addressed.

Although there have been many studies on the cooling performance of urban greenery^14^, there remain gaps in investigating EC in heterogeneous urban contexts at microscales^43^. The significant microclimatic variabilities and challenging urban conditions for vegetation growth point towards the need for better understanding the adjustment of urban trees to the local microclimate^29^ and the correlation between soil moisture content and EC that have not yet been fully investigated^13,14,44^. Consequently, more research is needed to determine the most effective spatial arrangements of urban greenery for cooling and outdoor thermal comfort benefits at local scales^14,45^.

Previous studies built a foundation on quantifying the human thermal comfort effects of the ecohydrology of soil-moisture-plant interactions^13,43,46,47^. However, most of the work related to soil moisture content and EC was conducted in rural contexts^43^. Many studies used a two-dimensional approach^48^, such as by means of satellite data^49^, although a three-dimensional approach is essential to capture the air flow and shading effects in dense urban environments^18^. Also, previous studies investigated the effect of irrigation of green infrastructure on ambient air temperature, reaching promising results on evaporative cooling^50,51^. To address limitations discussed in their work, it would be interesting to integrate mesoscale and microscale models to more comprehensively assess the potential of vegetation-based mitigation strategies, as well as to use thermal comfort indices, which are important to describe the human perception of its thermal environment.

Previous studies have also tried to separate the relative effects of vegetation shading and EC. However, it remains unclear how much each process contributes to urban cooling and how factors such as climate, urban form, vegetation type, and scale influence this^20,40^. Globally, research has quantified the full partitioning of ET^52^, or simply the proportion of ET from different sources^53,54^, without considering the effects of vegetation shading. Some studies managed to partition shading and EC for air temperature^20^ or both air and land surface temperatures^29^. In general, however, when comparing different cooling partitioning attempts, results are not consistent, showing dependence on initial and boundary conditions.

It is essential to address these gaps, as passive cooling coming from vegetation remains one of the most significant regulation ecosystem services for mitigating the impacts of climate change in urban areas^55^. The literature points to the need for more informed policy in heat mitigation and adaptation efforts^14,54^ and highlights that evidence on optimal vegetation configurations to improve thermal comfort is essential for aiding decision-making processes^44^.

Addressing the link between water and energy transport to quantify EC in urban environments remains underexplored, mostly due to the complexity of water dynamics in unsaturated soils^32^ and the high computational costs of microclimate models^2^. This complexity arises especially when taking into account different scales and varying many parameters, as studies need to account for multiple confounding factors under heterogeneous atmospheric and urban built environment conditions^20^.

Thus, the novelty of our study is to bring a comprehensive approach to evaluate the relative importance of urban tree cooling main drivers: vegetation amount, local climate, surrounding urban environment and soil moisture content (including irrigation), in order to partition the relative effects of tree shading and tree evaporative cooling on human thermal comfort. To address the research gaps and these challenges, we aim to answer three research questions: (*i*) how does the built environment and (*ii*) soil moisture content affect the cooling services provided by urban trees? And (*iii*) to what extent this cooling effect result from EC versus shading? Our study will contribute to provide guidance to more effectively evaluate whether urban trees and the irrigation of urban green areas are sufficient to address the current demands of heat mitigation and adaptation.

## Results

### Multi-scale modelling conceptualisation

**Fig. 2:**
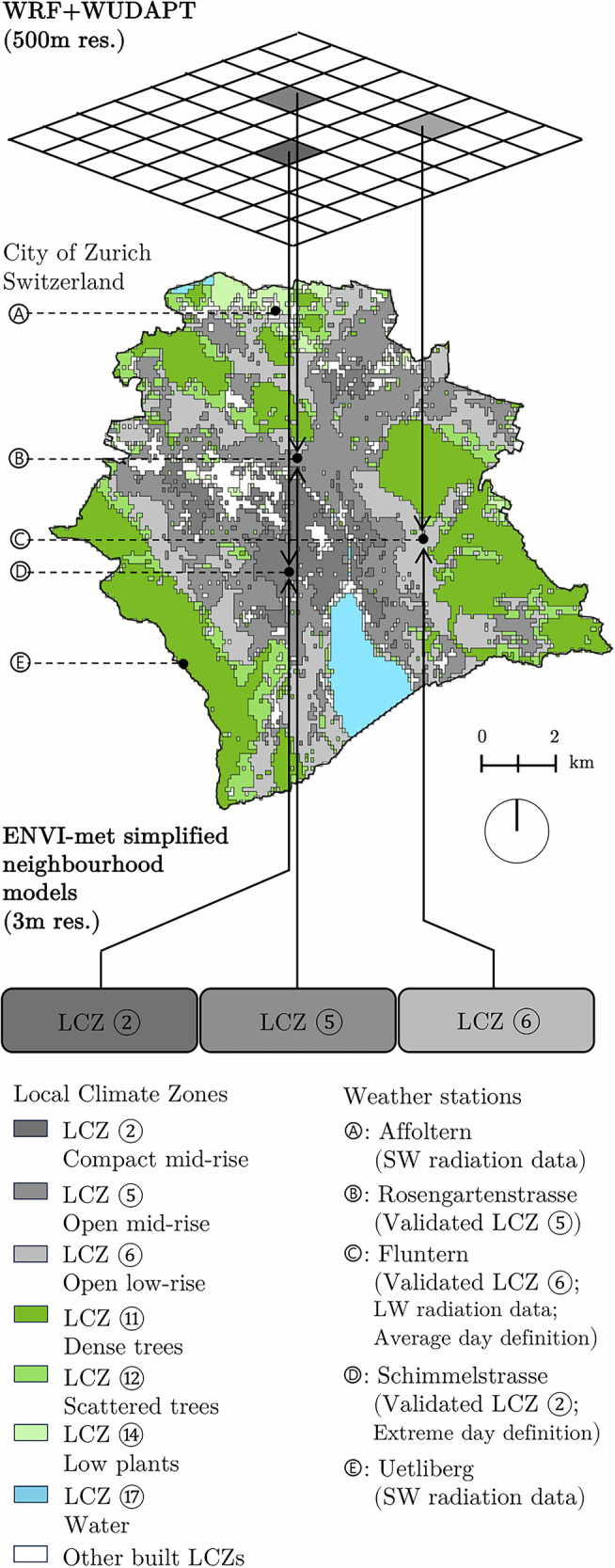
Map of local climate zones in the city of Zurich, Switzerland. WRF + WUDAPT produces gridded outputs, which serve as LCZ-specific weather forcing to the ENVI-met simplified neighbourhood models. These models were validated with weather stations within each respective LCZ. SW and LW mean shortwave and longwave radiation, respectively. Map is based on Demuzere et al. (2022)^83^.

To investigate the cooling potential of urban trees and their interaction with soil moisture levels under various urban forms, we employed a multi-scale modelling approach using ENVI-met (www.envi-met.com) and WRF (www.mmm.ucar.edu/models/wrf), see Fig. 2.

**Fig. 3:**
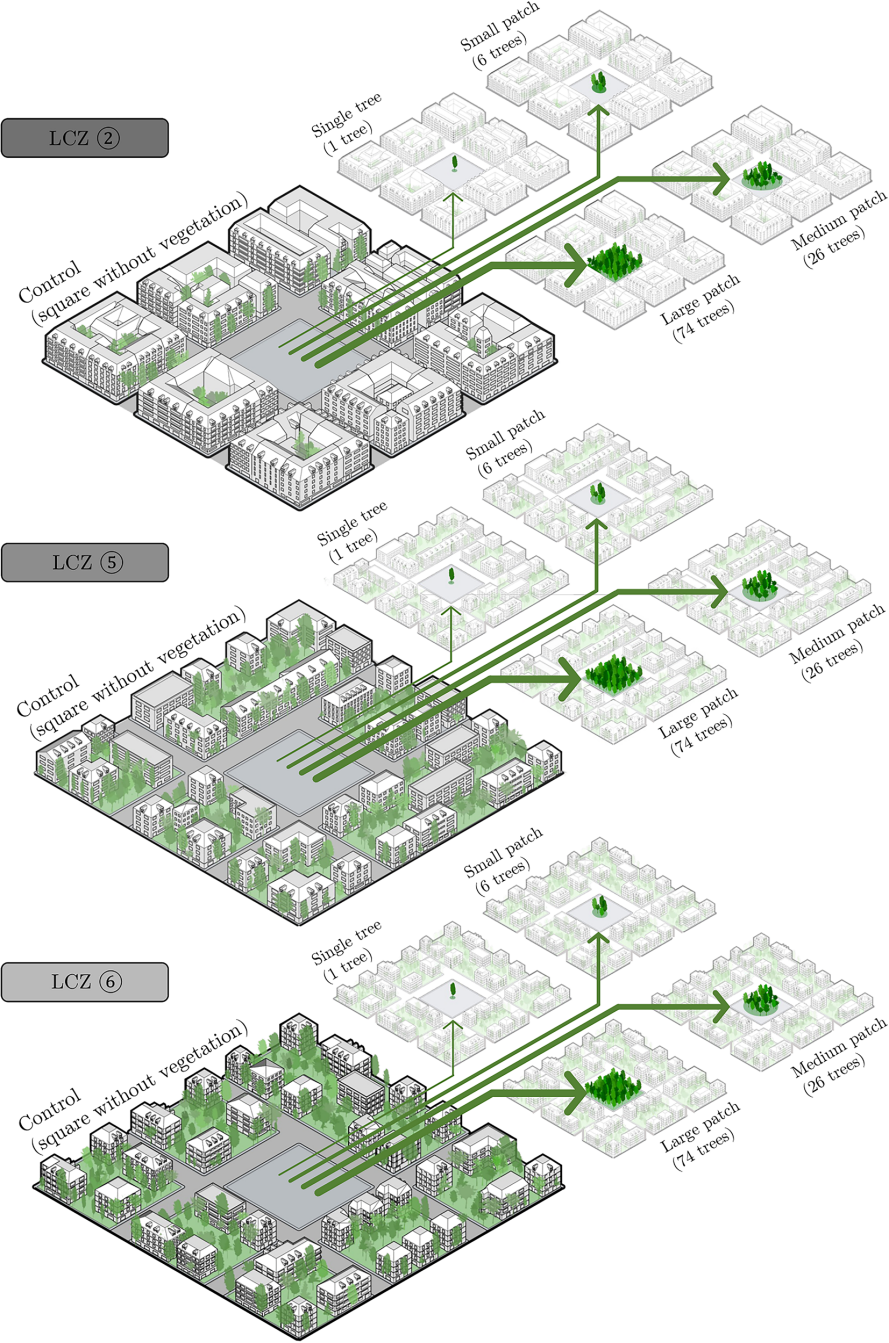
Illustration of the simplified neighbourhood models in ENVI-met, where variations occur in the central patch of the neighbourhood. Control scenarios are the models for each LCZ. The single tree, small patch of trees, medium patch of trees and large patch of trees are the experimental scenarios deriving from their respective control scenarios.

We divided the city of Zurich, in Switzerland, into Local Climate Zones (LCZ), and created a 'simplified neighbourhood model' representing each LCZ, see Fig. 3. These simplified models capture the thermal environment of their respective LCZ by incorporating average local built environment characteristics (more on Methods Section 'ENVI-met LCZ-specific simplified neighbourhood models'), as suggested by the literature^56^. We assume that the cooling effect of a tree patch added to the simplified neighbourhood model is representative of the effect that same patch would have across the corresponding LCZ. Although limiting in taking into account site-specific conditions, this approach reduces computational demand, allowing for the parameterisation of more variables and simulation of diverse scenarios, while still producing results that can be comparable relative to one another.

A key focus of this study is the influence of different soil moisture content scenarios on the cooling performance of urban trees under average and extreme summer conditions. The soil moisture ($\theta$) scenarios range from dry soil at the permanent wilting point (PWP, referred to as 'Dry'), to the soil moisture level where trees achieve maximum ET ('ETmax'), to fully irrigated soil at field capacity (FC, referred to as 'Irr') (more on Methods Section 'Relative soil moisture'). We simulate average and extreme summer conditions, which were selected based on historical climate data (more on methods Section 'Summer representative days').

**Fig. 4:**
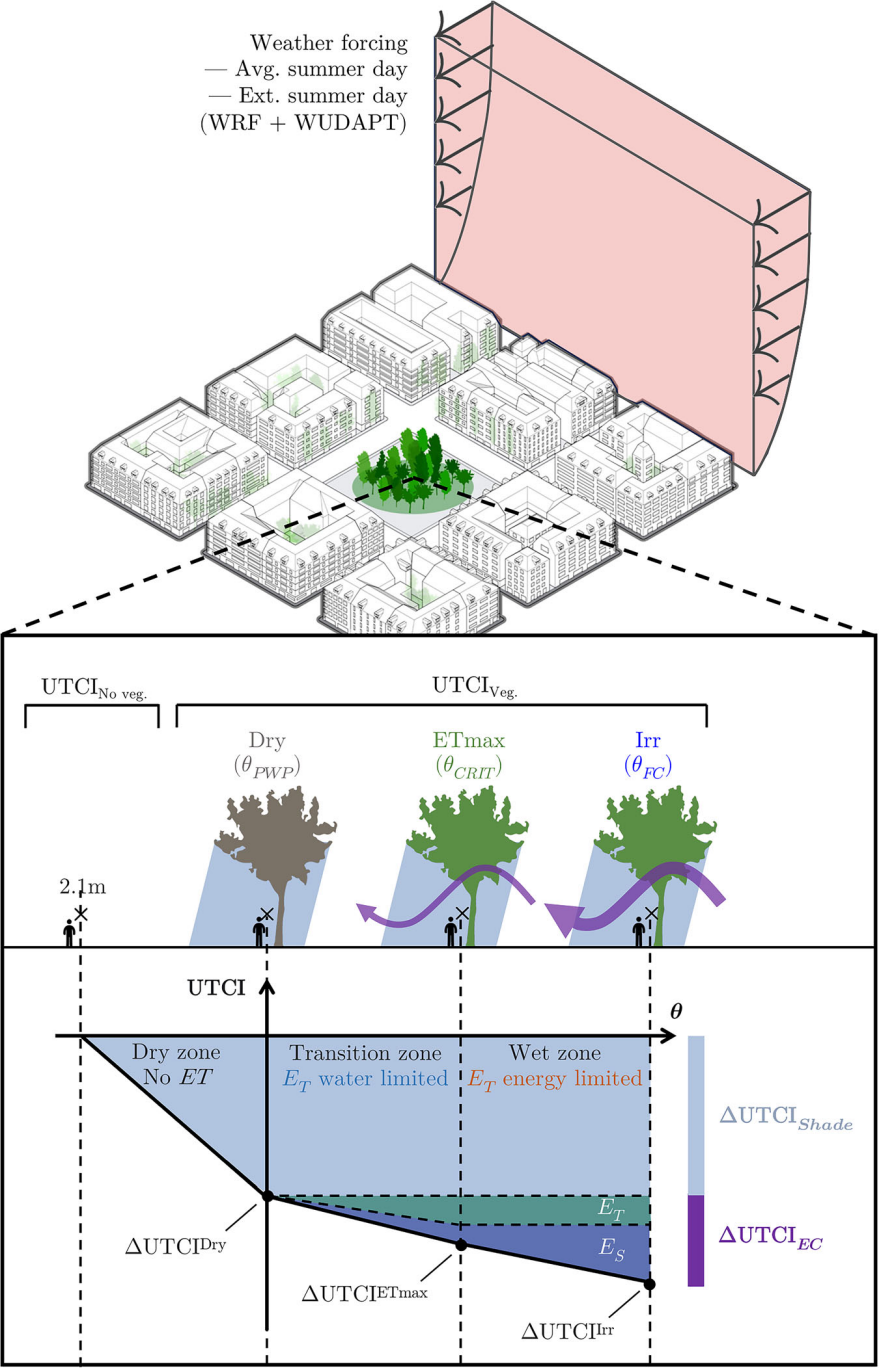
Simplified neighbourhood model (for LCZ ②) and soil moisture content scenarios. The image shows the lateral boundary conditions (average or extreme summer day) and the key performance indicator being measured: the UTCI at 2.1 m from the ground. ${\rm{\theta }}$ is the relative soil moisture content, ${{\rm{\theta }}}_{{\rm{PWP}}}$ the relative soil moisture at permanent wilting point (${\rm{\theta }}=0 \%$), ${{\rm{\theta }}}_{{\rm{CRIT}}}$ the incipient stomatal closure point or the maximum rate of plant ET without water limitations (${\rm{\theta }}=70\, \%$), and ${{\rm{\theta }}}_{{\rm{FC}}}$ the soil moisture at field capacity (${\rm{\theta }}=100\, \%$). ${{\rm{E}}}_{{\rm{S}}}$ is the direct soil evaporation, and ${{\rm{E}}}_{{\rm{T}}}$ the evaporation from plant transpiration. Notice that this relative soil moisture content notation is the one used by ENVI-met, where a fully oven dry soil ${{\rm{\theta }}}_{{\rm{OD}}}$ will have ${\rm{\theta }}=-100\, \%$.

The impact of varying tree patch size was also assessed, ranging from a single tree with a crown diameter of 9 m to a large patch of 74 trees with a total canopy diameter of 72 m (see Methods Section 'Vegetation'). This allowed us to determine the amount of greenery required to create vegetated outdoor heat refuges under varying climatic conditions. The physiological responses of trees were modelled to differentiate cooling effects derived from shading and evaporative cooling, with pedestrian-level thermal comfort evaluated through the Universal Thermal Climate Index (UTCI) (see Fig. 4) (more on Methods Section 'Assessment of results'). The data used in our framework can be seen at Methods Section 'Data', with an analysis on how to replicate this study for other cities.

### Validation of the simplified neighbourhood models

**Fig. 5:**
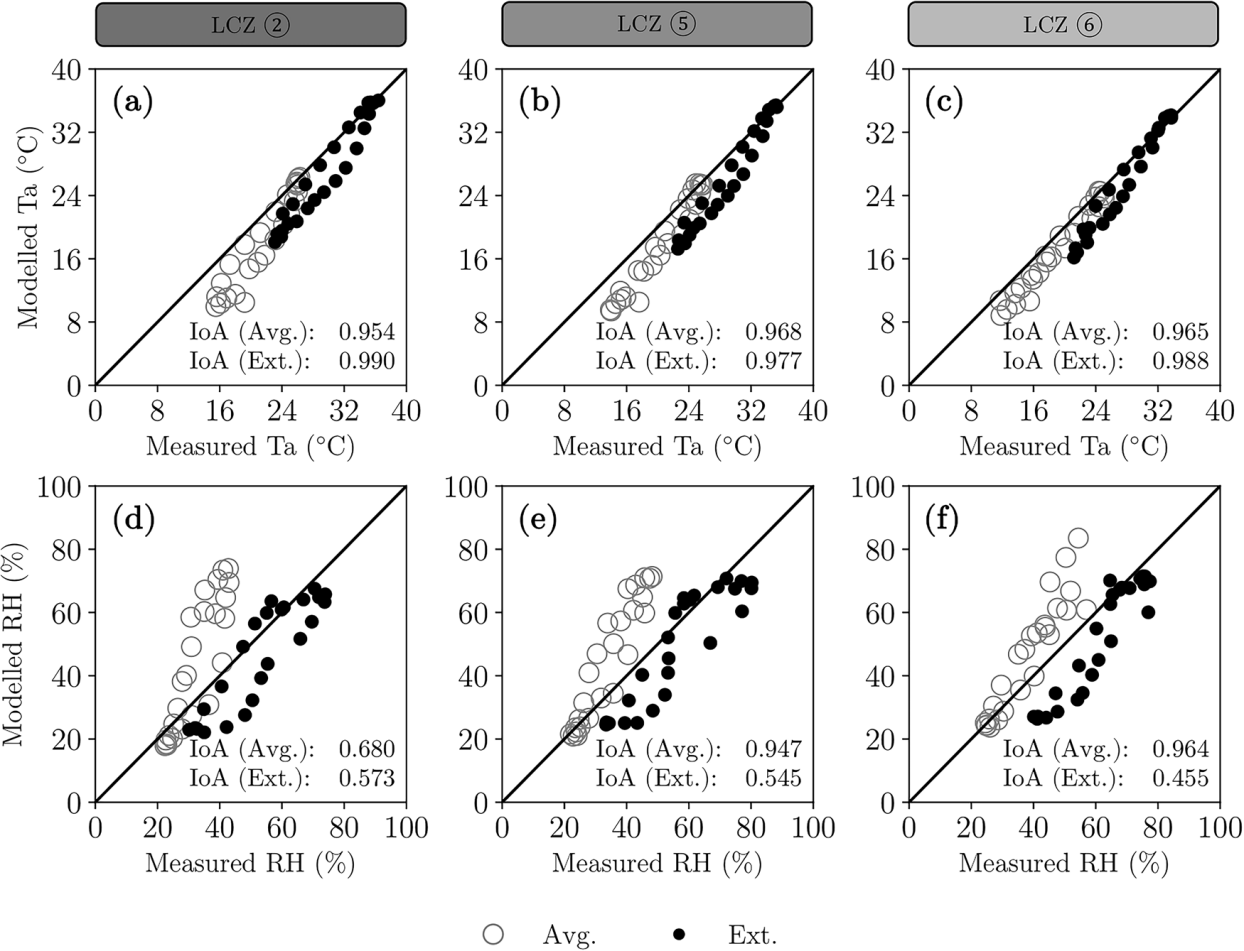
Comparison between modelled and measured air temperatures (Ta) and relative humidity (RH) in the centre of the simplified neighbourhood models in ENVI-met. Using WRF LCZ-specific forcing, for the average (Avg.) and extreme (Ext.) summer representative days, the plot shows a validation of air temperature (**a**–**c**) and relative humidity (**d**–**f**). IoA represents the Index of Agreement from 9 to 16 h only.

To ensure the simplified neighbourhood models accurately reflect the climate of their respective LCZs, each ENVI-met model was validated against measured weather data. These data were collected from weather stations pre-located by Switzerland’s official weather measurement network, located within each LCZ. We compared ENVI-met’s modelled air temperatures at 2.1 m above ground to those from weather station sensors positioned at 2 m, which was the closest possible match given the model’s resolution (see Fig. 5). The 10 cm height difference at this level results in only a marginal variation, making the comparison sufficiently accurate. We assume that a weather station within an LCZ can be a good proxy to the average air temperature and relative humidity values of that LCZ, which has inherent generalisation limitations.

The Index of Agreement shows that ENVI-met’s simplified models are more accurate in predicting daytime temperatures (9–16 h) than full-day temperatures (0–24 h) (further validation statistics are provided in the Supplementary Materials, Section S1). This discrepancy occurs because the lateral boundary conditions from the meso-climate model tend to underestimate the thermal capacity and inertia of the built environment due to its coarse spatial resolution and inherent simplifications^57^. As a result, denser built environments show greater mean errors. The same behaviour can be observed in relative humidity predictions, which our model underestimated during the night-time for the average summer day and overestimated during the day-time for the extreme summer day. The simplified neighbourhood models tend to yield better relative humidity results during average summer days and within less dense and more vegetated LCZs. This discrepancy occurs because of inherent limitations to our modelling approach: we are measuring relative humidity in the centre of the simplified neighbourhood model and in the control scenario, a place without vegetation, while our measured data comes from areas in the city where vegetation is really close by.

Despite these limitations, the simplified models have a good enough air temperature alignment with measured data, particularly during peak daytime temperatures (9–16 h), the main focus of this study. Although they systematically underpredict air temperatures, especially at night, and underpredict relative humidity in extreme events, the models remain sufficiently accurate for our application which focuses on daytime conditions. We expand on the impact of these limitations in the discussion section.

We also acknowledge concerns raised by Crank et al. (2018)^58^ that ENVI-met is not fully grid-independent and remains partially proprietary. To address these issues, we adopted the finest feasible resolution given our computational constraints, validated our simplified neighbourhood models against local LCZ weather stations, and explored parametric scenarios focusing on the main variables we were concerned of. As a result, any minor biases linked to grid sensitivity do not overshadow our principal findings on urban tree cooling efficacy. Furthermore, even without full access to the model’s source code, ENVI-met’s core simulations remain sufficiently reliable for our study objectives. Each scenario required about four hours to run on a standard personal computer (eight cores), demonstrating a good balance between resolution and computational efficiency.

### Influence of soil moisture in tree cooling

**Fig. 6:**
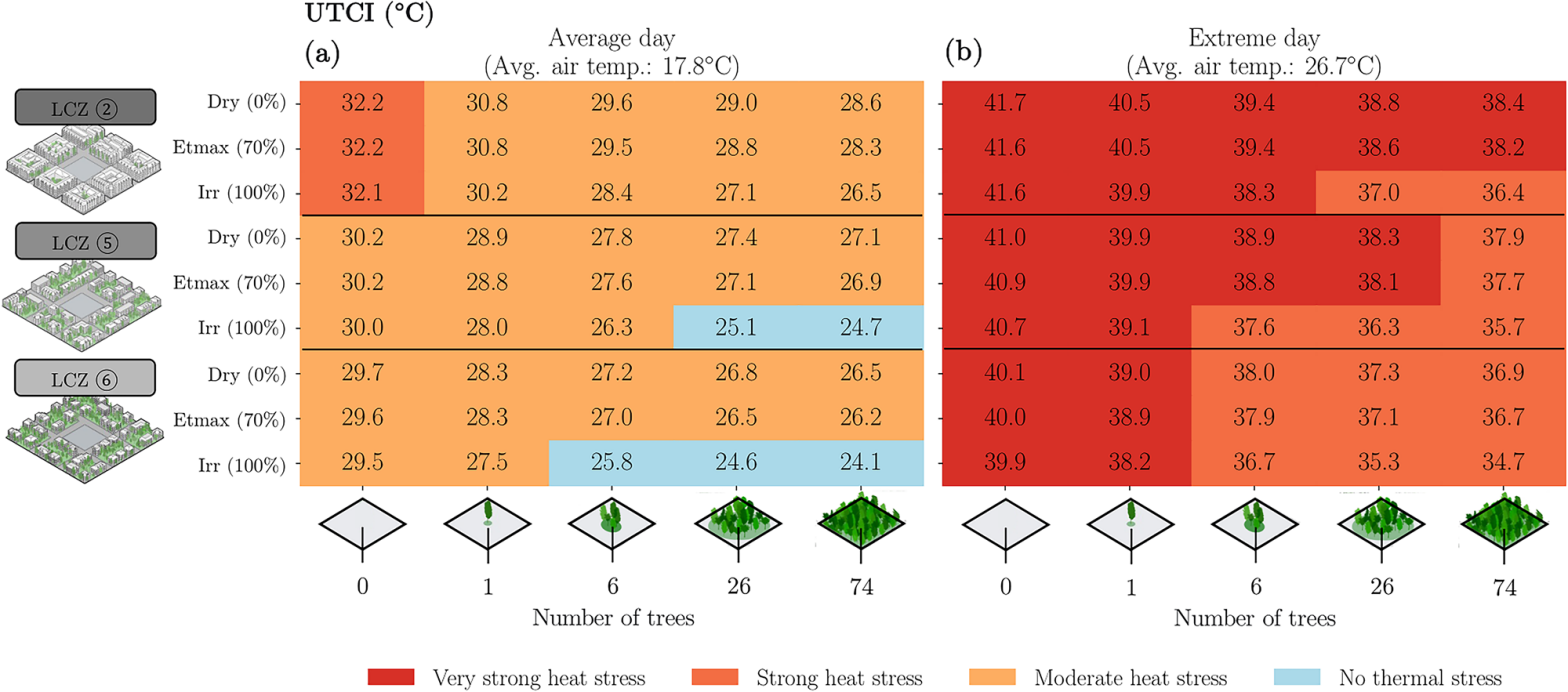
Modelled UTCI results. Thermal comfort (UTCI) is modelled at 2.1 m above ground in the centre of the model domain, at 14 h (peak daily air temperature), for the average (**a**) and extreme (**b**) summer day scenarios, for the different number of trees (*Carpinus betulus*), LCZ and relative soil moisture contents.

The calculated UTCI indicates that, even if under greenery cover, a person would be in most cases under some level of thermal stress during average and extreme summer days (Fig. 6). The absence of thermal stress conditions could be observed in the LCZ ⑤ (open mid-rise) during an average summer day and if the person was under a tree patch with 26 trees or more. This number was reduced in LCZ ⑥ (open low-rise), where only 6 trees would be enough to achieve a no thermal stress condition. Notably, reaching this condition of thermal comfort happened only in the irrigated (Irr) scenarios. The results showed clearly that increasing the relative soil moisture content to field capacity, using active irrigation, contributes substantially to lowering the thermal discomfort. For the extreme day, a ‘no thermal stress situation’ could not be reached, although some improvements from very strong heat stress to strong heat stress were achieved.

**Fig. 7:**
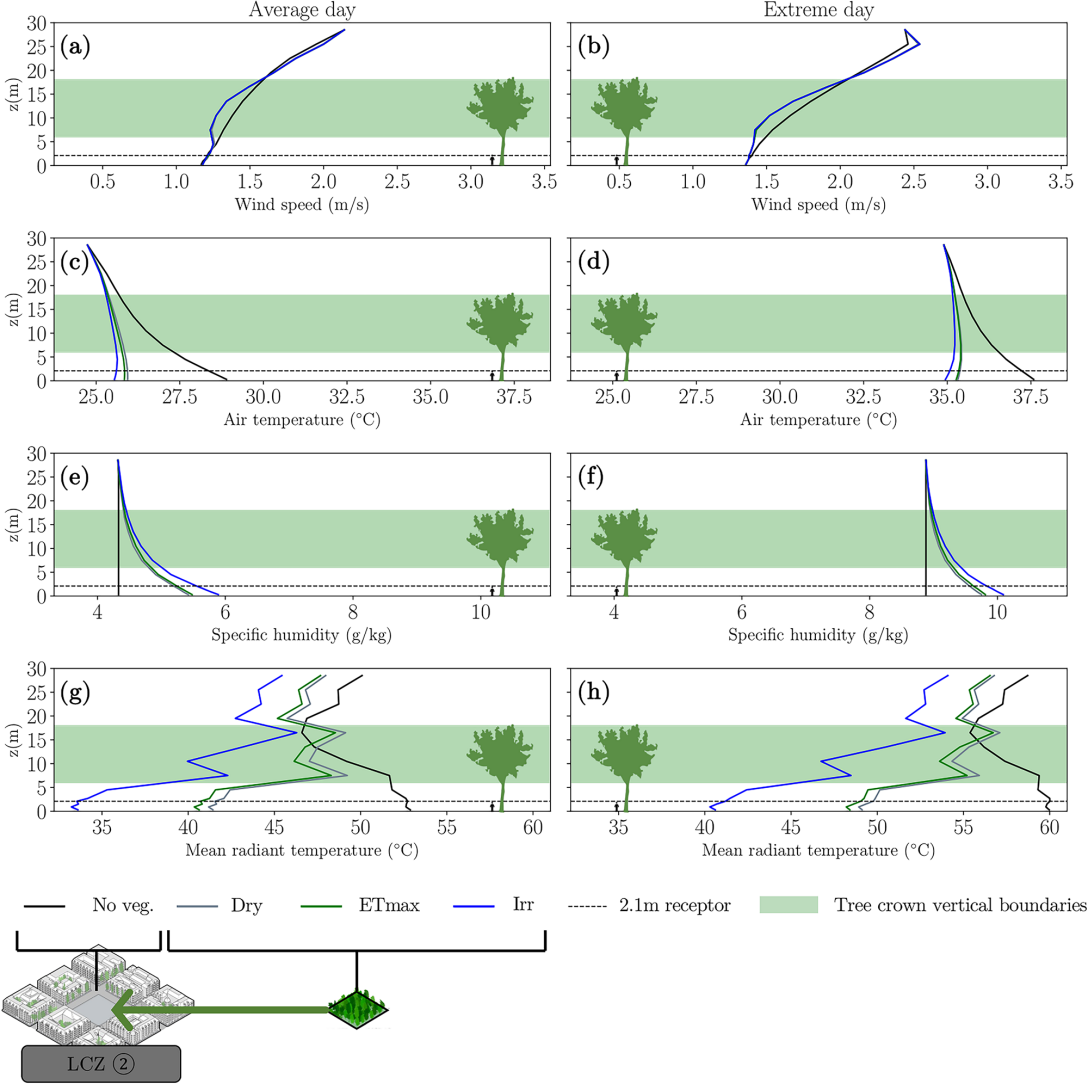
Vertical profiles at the centroid of the model at 14 h for LCZ ② when adding a large patch of greenery. For both average and extreme summer day scenarios, we show the vertical profiles of wind speed (**a**, **b**), air temperature (**c**, **d**), specific humidity (**e**, **f**) and mean radiant temperature (**g**, **h**). Curves indicate different soil moisture content scenarios: dry soil at permanent wilting point (${{\rm{\theta }}}_{{\rm{Dry}}}$); soil that reached maximum ${{\rm{E}}}_{{\rm{T}}}$ (${{\rm{\theta }}}_{{\rm{ET}}\max }$); irrigated soil at field capacity (${{\rm{\theta }}}_{{\rm{Irr}}}$), reaching maximum ${{\rm{E}}}_{{\rm{S}}}$.

In the Dry and ETmax scenarios, we found that the main variables governing UTCI (wind speed, air temperature, specific humidity and mean radiant temperature) vary in a similar way along the vertical profile of the model centroid (see Fig. 7). However, for the Irr scenario, the addition of water creates a large impact, especially in the mean radiant temperature. This impact is greater the closer it gets to ground level. Which indicates that, if there is no soil water replenishment (irrigation), most of the available water in the soil is used up by canopy transpiration in ENVI-met. This, however, may not be true in reality: parts of the superficial soil can be evaporating at the same time as deeper soil is providing water for root uptake. As such, in ENVI-met, temperatures at canopy height are the ones mostly affected by evapotranspiration, with little cooling impact observed at pedestrian-level. On top of that, across all scenarios no significant cooling advection effect was detected. This implies that the cooling benefits were primarily localised directly beneath the tree canopies, with only marginal cooling effects on tree surroundings.

### Further increasing the number of trees

**Fig. 8:**
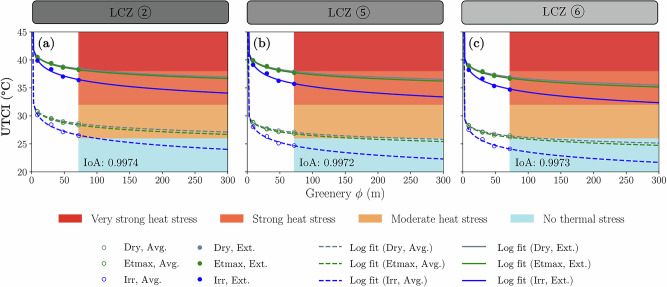
Projected progress of thermal comfort as greenery increases relative to the control scenario without trees. Thermal comfort (UTCI) is modelled at 2.1 m above ground in the centre of the model domain at 14 h (peak daily air temperature), for LCZ ② (**a**), ⑤ (**b**) and ⑥ (**c**). Markings on white background are modelled data, and lines are a natural logarithmic function fit projection. The coloured background with UTCI categories shows the area where greenery diameter is higher than 72 m and thus shows only the predicted log fit. The average index of agreement (IoA) per LCZ is marked in each plot (further details on fitting in the Supplementary Materials, Section S2).

As shown in Fig. 6, the no thermal stress condition was rarely achieved, raising the question of whether increasing the amount of greenery could be a solution. When plotting the modelled ΔUTCI against greenery diameters across various soil moisture contents and summer scenarios, the results suggest a plateau, illustrated by the logarithmic curve fit in Fig. 8.

While infinite greenery theoretically leads to a ΔUTCI of −∞, in practical terms, a 300 m tree patch represents the maximum greenery addition within the constraints of LCZ classification at a 100 m resolution. Also, at some point, there is simply not enough space in the urban environment. At this 300 m diameter, Dry and ETmax soil moisture scenarios show a maximum cooling of about −5 °C UTCI, while the irrigated (Irr) scenario achieves up to −8 °C UTCI. On average for all LCZs and summer representative days, a 10% increase in tree area represented a cooling of $0.048\,\pm 0.003$ °C UTCI for the Dry and ETmax scenarios and a cooling of $0.081\,\pm 0.002$ °C UTCI for the Irr scenario. This highlights the effectiveness of irrigation in reducing thermal stress.

However, for the highly urbanised scenario (LCZ ②), a ‘no thermal stress’ situation remains unreachable through greenery alone, even with irrigation. During extreme events, a ‘no thermal stress condition’ was never reached, even with large green areas and full irrigation. Notably, during heat waves, even dense vegetation outside cities may not prevent thermal stress, making it even less likely for urban green areas affected by the urban heat island effect to do so.

### Cooling from shade versus cooling from evaporation

**Fig. 9:**
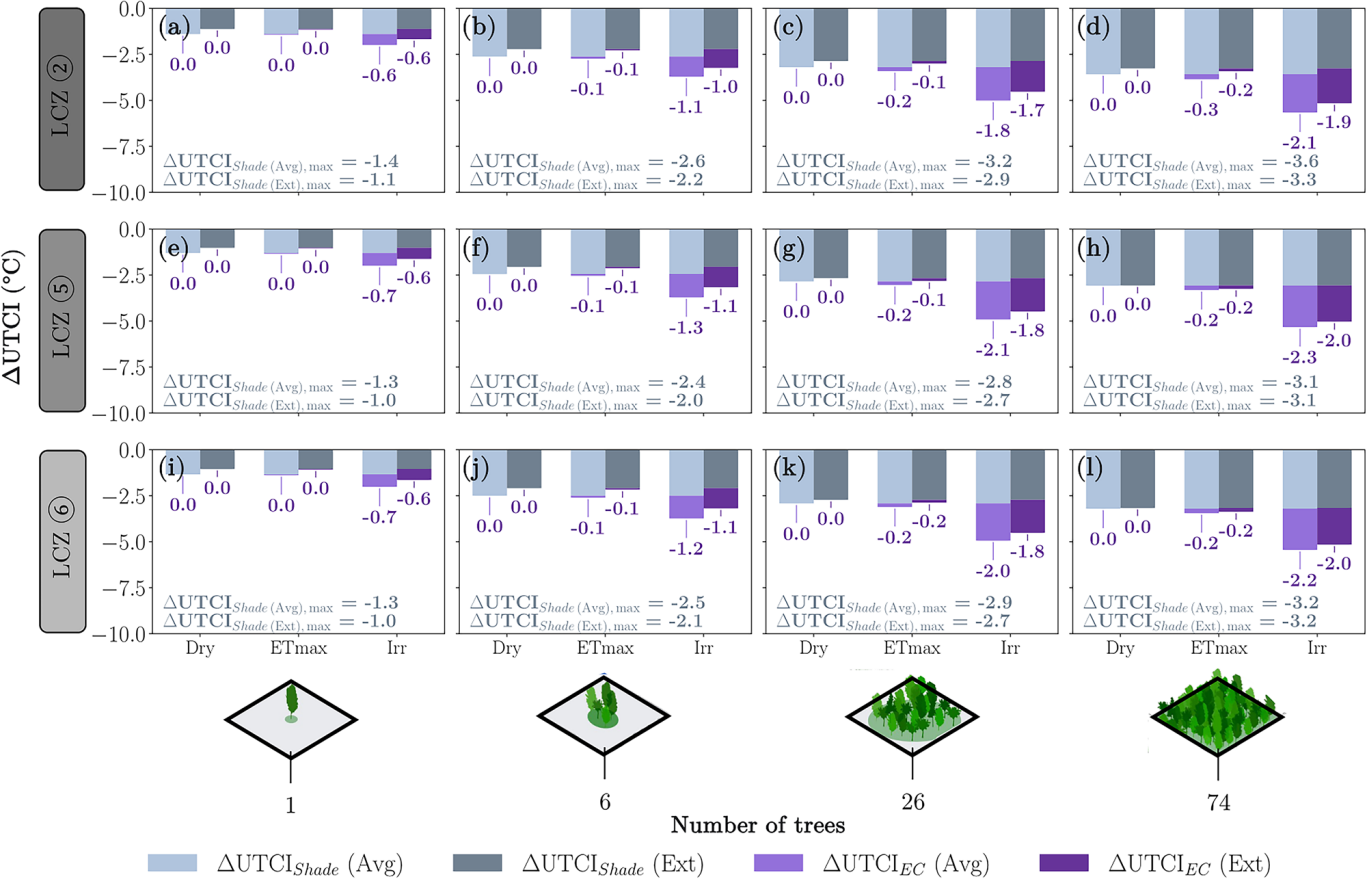
Modelled ΔUTCI results. The delta thermal comfort (ΔUTCI) is modelled at 2.1 m above ground in the centre of the model domain at 14 h (peak daily air temperature) for the average (Avg.) and extreme (Ext.) summer day scenarios, for the different number of trees (*Carpinus betulus*), LCZ and relative soil moisture contents. First row of panels is for LCZ ② (**a**–**d**), second row for LCZ ⑤ (**e**–**h**) and last row for LCZ ⑥ (**i**–**l**). Annotations on the bottom left corner of each subplot show the ΔUTCI_Shade_ values, which are constant for Avg. and Ext. scenarios, and the annotations below each column show the ΔUTCI_EC_ values in °C of UTCI.

When decoupling the effects of shade and EC, the highest contribution of EC was observed not during ETmax, but in the irrigated soil scenario (see Fig. 9). This indicates that direct evaporation from bare soil significantly enhances thermal comfort at pedestrian height, as the wet substrate acts as a heat sink. Additionally, it is important to emphasise that vegetation performed better in average summer conditions than in extreme summer conditions. Once leaf temperature exceeds a certain threshold, stomata begin to close, which directly limits ET and, consequently, EC, thereby reducing the cooling effectiveness of trees during extreme heat (further details are available in the Supplementary Materials, Section S3). This aligns with recent studies that also show models can systematically overestimate the cooling effect of trees during heat waves^59^.

### Water volume needed for irrigation

**Fig. 10:**
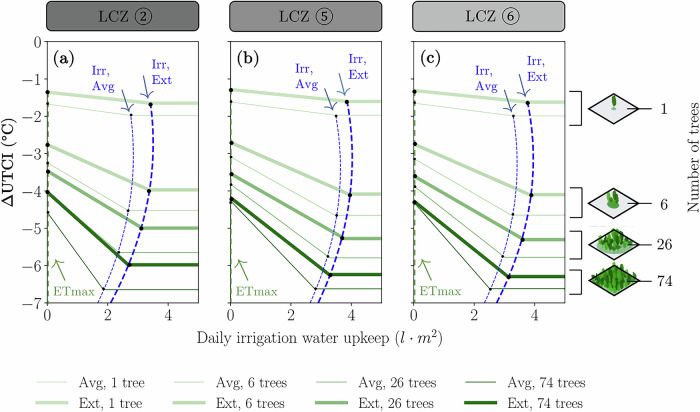
Modelled daily irrigation water upkeep to maintain the trees at field capacity. Calculated by estimating the water quantity needed in order to match the water lost by tree transpiration and soil evaporation per unit area. Panels show LCZ ② (**a**), LCZ ⑤ (**b**) and LCZ ⑥ (**c**). At daily irrigation smaller than the Irr scenario boundaries (depicted in dashed lines for Avg. and Ext. scenarios), the soil would start to lose water and reach the behaviour of the ETmax scenario. If water input continues smaller than the boundaries, the conditions can go all the way down to the Dry scenario, not depicted here, but which are roughly similar to ETmax as seen in Fig. 6.

Maintaining the soil at field capacity seems to be a determining factor in providing outdoor vegetated cooling refuges. For the soil profile used in our models, the volumetric water needed to take one m3 of that soil from permanent wilting point to ETmax is 68.9 l⋅m^−3^. To further take it from ETmax to field capacity, 29.52 l⋅m^−3^ would be needed. After that, Fig. 10 shows the volume of daily irrigation that would be needed to upkeep relative soil moisture content at field capacity (Irr) scenarios and its influence on ΔUTCI.

These irrigation volumes were estimated by integrating the changes through time in soil volumetric water content along the plant root depth, rather than simulating an explicit irrigation event. However, in real-world applications, the choice of irrigation system, the depth of irrigation, and the irrigation timing are critical factors, as these factors can influence the effective water replenishment and subsequent cooling outcomes. Our results apply to the soil types in our models (see data availability section for more information about the soil profiles), as actual pore saturation also depends on soil physical and chemical properties and required irrigation depths.

## Discussion

Our study demonstrates that soil moisture significantly impacts thermal comfort, particularly when irrigation raises soil moisture above the ETmax threshold. Irrigation improved thermal comfort during average summer days, reducing thermal discomfort by several UTCI degrees, as shown in LCZ ⑥, where six irrigated trees decreased UTCI from 29.5 °C to 25.8 °C (Fig. 6). The water required to maintain soil moisture at field capacity ranged from 2 to 4 *l*⋅m^−2^. While irrigating existing trees offers short-term relief, aligning with previous studies, long-term strategies require strategic tree planting^48^. This points to the direction of strategic spatial planning for tree locations based on cooling performance, possibly exploring various water sources and qualities for irrigation to optimise water reuse.

However, we found that greenery alone is not sufficient during extreme heat days in Zurich. For LCZ ⑥, without irrigation, the largest possible green area was required to mitigate heat stress (Fig. 8). Even with optimal irrigation and maximal tree coverage, the cooling effect capped at −8 °*C* ΔUTCI. Here we also note that our models systematically underpredicted relative humidity during day-time conditions in the extreme summer representative day. This means that possibly our results underpredict heat stress during extreme days, indicating that UTCI values would probably be even worse than we modelled during heat waves. Our findings also show that larger tree patches, however, reduced irrigation needs by conserving soil moisture through shading, which again is aligned with previous studies^46^.

Previous studies have shown that the overall cooling contribution from vegetation varies with species and design^29,60^. Many urban tree species with high drought resistance are not well understood in terms of their cooling dynamics^20^. The literature points to trees with anisohydric behaviour, for example, that could continue to provide cooling through transpiration even under reduced leaf water potential^55,61,62^. From our results, however, thermal comfort cooling came mostly due to direct soil evaporation and tree shading, so we expect that trees with varying evapotranspiration regimes would not substantially affect the results. Future research can focus on evaluating a wider range of tree species with such traits to test this hypothesis. We should note, however, that if a soil is kept at field capacity, vegetation will have a dynamic behaviour and will grow more, resulting in higher evapotranspiration contributions. Our results apply to mature Hornbeam trees, whose cooling performance takes years to reach the levels described in our study due to the time required for full establishment^63^. Future work can explore the dynamic cooling effect of trees to assess how their impact on pedestrian thermal comfort evolves throughout their lifespan.

In most cases, tree shading was clearly the primary cooling mechanism, providing from 1.0 to 3.6 °C ΔUTCI. In irrigated scenarios, EC provided from 0.6 to 2.3 °C ΔUTCI, with respectively at least 0.6 to 2.1 °C ΔUTCI coming from direct soil evaporation (Fig. 9). This shows that, for high water availability scenarios, EC and shade can have similar orders of magnitude. We found a substantial influence of soil evaporation, particularly near the ground where thermal comfort is measured, confirming the findings from earlier studies^45,64^. We found no comparable studies that partition thermal comfort between direct soil evaporation and tree transpiration. Rather than expressing the contributions as percentages of total ΔUTCI, we report the absolute cooling differences. This approach avoids oversimplifying the complex, nonlinear dynamics of energy transfer. Importantly, a higher ΔUTCI not only signifies larger cooling effect but also increases the likelihood of shifting between UTCI categories, thereby more effectively enhancing thermal comfort.

The first main implication of our work is related to water management. While keeping soil above field capacity enhances cooling, maintaining some capacity of water retention can also contribute to flood risk management, indicating a trade-off^65^. Intelligent irrigation systems, guided by weather forecasts, could optimise soil water content for both heat mitigation and water management, e.g., irrigating during heat days and stopping irrigation for soil retention capacity recharge to accommodate rainwater during precipitation events. Maintaining soil at field capacity strikes a good balance between water and air availability. Good drainage is key to avoiding waterlogging, which can deplete oxygen around roots under excessive irrigation. Frequent, light irrigation can help keep soil moisture near field capacity while allowing air exchange, reducing the risk of oversaturation^66^.

Second, our findings also have implications for the use of mulching. While mulching can significantly reduce direct soil evaporation, which benefits plants and local biodiversity by conserving water, our results show that soil evaporation plays a key role in improving thermal comfort, indicating a trade-off. Intelligent mulching management is essential, such as ensuring that irrigation is not applied beneath the mulch but is intercepted by it, allowing for evaporation. Although bare soil is often impractical in urban settings^29^, tree grates would offer a practical solution, promoting direct soil evaporation while maintaining pedestrian and vegetation safety and preventing soil compaction.

Finally, our third main finding relates to climate mitigation and adaptation strategies. Trees should be prioritised for new green spaces aimed at providing local heat mitigation and shelter during average summer days. However, for extreme heat events, additional measures, such as temporary shelters with active cooling or using existing public spaces as heat refuges, may be necessary^35^. With future increases in extreme weather events and decreased soil moisture globally^67^, a better understanding of soil moisture-plant interactions will be critical for addressing heat stress and drought challenges^43^.

Our study’s limitations relate to the high computational demands of microclimate simulations, which prevented us from testing different tree species and broader geographic applicability, e.g., in other Köppen-Geiger climate zones. Although this study focuses on Zurich (Switzerland), which has a high heat adaptation need, the methodology is generalisable and provides a replicable framework for assessing the influence of soil moisture and the built environment on urban greenery’s cooling performance of other climatic regions. Future research can apply these methods in different cities and climates to explore how varying conditions impact tree evaporative cooling.

The outcomes found in our study could also be influenced by vegetation management (such as pruning), plant choice and different soil types, pointing towards the risk of oversimplification: our results are relevant for Zurich, but local conditions should be taken into account to derive our results to other areas. For different climates where e.g., air temperature or relative humidity dynamics are different than those in Zurich, other relations between shading and evaporative cooling could be found. Additionally, improving the model’s accuracy for nighttime heat dynamics and indoor environments, along with assessing thermal comfort for diverse metabolic rates and heat-sensitive groups, would further enhance the applicability of the presented approach.

Although validating only air temperature and relative humidity in the control scenarios (without trees) does not by itself confirm ENVI-met’s representation of shading or ET, we strengthened confidence in these processes by comparing the sanity of different parametric scenarios and examining vertical profiles. While Liu et al. (2021)^68^ highlight limitations in older ENVI-met vegetation routines, recent developments (e.g. Sinsel, 2022^69^) incorporate refined ray-tracing for plant canopies, differentiated leaf sunlit/shaded processes, and improved biophysical modules. Using these updates (version V5.6.1 2024), we observed good agreement between our findings and the expected tree shading and ET behaviour, suggesting that ENVI-met reliably captures the critical mechanisms of urban tree cooling relevant to this study. Future work should include leaf-scale flux measurements as well as below tree crown measurements of variables relevant to thermal comfort (such as air temperature, relative humidity, wind speed and MRT) to further validate our findings and independent validations of ENVI-met’s tree model should also be performed.

The role of water management in maximising the cooling benefits of urban greenery is crucial. Our findings emphasise the need for comprehensive urban planning strategies that integrate water management with passive cooling solutions to mitigate urban heat. The synergy between irrigation and greenery offers both immediate and long-term solutions to reduce urban heat stress. As climate change continues to increase the frequency and intensity of heat stress days, integrating these approaches will be vital for protecting public health, reducing heat-related illnesses, and ensuring sustainable urban environments.

## Methods

To model tree cooling in a way that is replicable in other cities worldwide while not being too computationally demanding, we built a framework that combines numerical climate models and geographic information systems. We tested this framework for the city of Zurich in Switzerland, which is under a temperate climate without a dry season and with a warm summer (Cfb) according to the Köppen Geiger classification^70^. The choice of Zurich lies in its information availability and built environment heterogeneity: it is a municipality with a large network of weather stations with open weather data, as well as having accurately documented building characteristics and a wide range of building morphologies and land uses, while not having the land cover and land use complexity of a megalopolis.

### ENVI-met LCZ-specific simplified neighbourhood models

We modelled the cooling performance of trees using ENVI-met V5.6.1 2024, a widely utilised microclimate model based on simplified Computational Fluid Dynamics principles^71^. The model incorporates weather forcing as a lateral boundary condition and solves the non-hydrostatic incompressible Navier-Stokes equations for each grid cell at every time step, generating a three-dimensional representation of mean turbulent air flow, considering heat sinks and sources from surface-vegetation-air interactions^36^. Weather forcing input can be derived from a meso-climatic model or point scale measurements^14^. Additionally, the model outputs climate-dependent variables, such as surface and air temperatures as well as thermal comfort, which expresses a person’s perception of their surrounding thermal environment^36^.

The LCZ classification, as described by Stewart and Oke (2012)^72^, allows for a thermal description of the built environment according to its surface structure, surface cover, surface materials and local human activity. While there are limitations in consistently applying the LCZ classification system across different urban environments, and further refinement may be necessary for specific local contexts, it offers a standardised framework for urban temperature observations, saving on computational time^73^. Instead of simulating large areas of Zurich to determine the effect of vegetation in various built environments, we synthesised extensive areas with similar thermal conditions into what we term 'simplified neighbourhood models,' based on local LCZ classifications.

These models are not based on the actual morphology of any existing area within an LCZ but are the result of weighted averages of building footprints, building heights, street widths, and block sizes within each LCZ. All of these parameters are critical for accurately defining local access to solar radiation, wind flow regimes and, thus, describing local thermal environments^74^. The use of idealised neighbourhood models is common in many other studies to enhance the generalisability of results^68^.

To account for the heat island effect, the lateral boundary conditions used in the simplified neighbourhood models had to be LCZ-specific. These boundary conditions were generated using the meso-climate Weather Research and Forecasting (WRF) model, a non-hydrostatic compressible model, initially designed for weather prediction but widely applied as a regional climate model^75^. We ran the WRF model for Zurich and its surroundings with a 500 m resolution over a 30 × 30 km domain. The output of WRF is a prediction of local weather conditions for every pixel, given input conditions (see Table 1 in ‘Data’ section for more details). From this WRF output, single-pixel data were extracted for each LCZ at the location of its corresponding local weather station. For each reference pixel, WRF outputs of radiation (longwave and shortwave), air temperature, relative humidity, and wind speed at 2 m height over 24 h were used directly as weather forcing in ENVI-met. This approach provided LCZ-specific lateral boundary conditions based on WRF-derived reference pixels. For the detailed boundary conditions used, refer to our data availability section. By using this method and accounting for the broader influences of the built environment on local microclimates, we reduced the ENVI-met domain size to 400 metres by 400 metres with a 3 m resolution in the simplified neighbourhood models.

**Table 1:** Datasets, databases, tools, research products and standards incorporated in this framework for weather forcing, model morphology and vegetation

Input	Source	Description	Coverage	If case-specific, what option of global dataset exists?
Weather forcing				
WRF historical analysis data	NCEP (2015)^80^	Input data for WRF. The geographic static data used was simply the highest resolution of each mandatory field, available at the model website	Global	—
WUDAPT to WRF (w2w) tool	Demuzere et al. (2022b)^81^	Incorporates the LCZ classification in WRF. This tool allows for the WRF model to have a more diverse classification of the urban fabric (up to 11 built classes), generating more accurate climate results within urban areas	Global	—
Climatology	*climate.onebuilding.org*	The climatology provided by Climate One Building was used to find the average summer representative day, which is based on a combination of data from ECMWF ERA5 reanalysis, US NOAA’s Integrated Surface Database and local weather stations, in this case Zurich’s Fluntern weather station	Global	—
Roughness length	Dörenkämper et al. (2020)^82^	Models the wind speed profile. We used the New European Wind Atlas and adopted a roughness length ${z}_{0}=0.5$ for all LCZs	Local (continental)	The Global Wind Atlas provides coarser estimations (*globalwindatlas.info*)
LCZ-specific weather stations	MeteoSwiss measuring network	The extreme day was selected from the weather station in Schimmelstrasse, which is the one within the denser parts of the city and thus more sensitive to heat waves	Local (federal)	If no weather station is available for each LCZ, validate WRF only with a surrounding weather station (e.g. closest airport)
Urban morphology				
Local Climate Zones	Demuzere et al. (2022a,c)^83,84^	Global map of Local Climate Zones	Global	—
Built environment geometry	Canton of Zurich: 'Vektor-Übersichtsplan (OGD)' - 2024	Data on building footprints, building heights, street widths and block sizes. Relevant to build the morphology of each LCZ simplified neighbourhood model.	Local (cantonal)	*openstreetmap.org* (street width would have to be calculated)
Soil profile	Agroscope: 'NABODAT Swiss Soil Dataset' - 2022; Swisstopo: 'Mächtigkeitsmodell des Lockergesteins' - 2021 and Twarakavi et al. (2010)^85^	Public data on federal level of soil profile composition and average depth before bedrock. An average soil profile among all LCZs was selected and classified according to the ternary sand-clay-silt diagram. Relevant to solve the soil profile input in ENVI-met	Local (federal)	Data on soil profiles can be found at *soilgrids.org*
Building materials	Canton of Zurich: 'Gebäudealter' - 2019 and Schwab et al. (2016)^86^	Research data on average building stock materials and layers thickness for roofs and walls per historical period in Switzerland (pre-war, interwar, post-war or high economy periods). This was crossed with building stock age data on cantonal level. Relevant for generating an estimated average combination of building materials specific to each LCZ simplified neighbourhood model	Local (research and cantonal)	Query data locally from experts or from local standards and construction guidelines
Pavement materials	City of Zurich: 'Dimensionierung Strassenoberbau TAZ' - 2012	Municipal level norm regulating standards for carriageway and sidewalk pavements. Cross sections were estimated to be the same for both cases. Relevant to estimate an average pavement material to be applied to any impervious area in the simplified neighbourhood models	Local (municipal)	Query local norms and standards
Bedrock profile	Pavoni (1957)^87^	Research data on Zurich’s bedrock profile composition, relevant to solve the bedrock profile input in ENVI-met	Local (municipal)	Query local geological datasets
Vegetation				
Tree stock	City of Zurich: 'Bauminventar' - 2021	Selection of the most common tree species in the city	Local (municipal)	Global Urban Tree Inventory (GUTI, vers. 1.0)

### Relative soil moisture

Varying the relative soil moisture content ($\theta$) is the most relevant parameterisation for understanding the impact of water on the thermal comfort improvements provided by urban trees. However, as indicated by Rodríguez-Iturbe and Porporato (2005)^30^, ET does not vary linearly with $\theta$. For high enough $\theta$ to allow for the normal course of plant’s physiological processes, ET is mainly a function of plant type, soil and climate, being a constant ETmax, independent of $\theta$. When below a threshold ${\theta }_{{CRIT}}$, the point of incipient stomatal closure, the plant transpiration starts reducing and $\theta$ becomes a key determinant factor of actual ET^76^. Reducing soil water availability until wilting point ${\theta }_{{PWP}}$, impacts the root water uptake. After reaching the permanent wilting point (PWP), water is only lost by soil evaporation. About 99% of the water xylem supply taken up from soil goes to transpiration and only 1% goes to metabolic activities, showing the importance of water flux in plants for temperature regulation^76^. A sufficient supply of water to leaves is, thus, relevant for avoiding permanent leaf heat damage from cavitation and excessive water loss^77^.

Three different points of $\theta$ are, hence, relevant to characterise the relationship between water availability and ET: ${\theta }_{{PWP}}$, ${\theta }_{{CRIT}}$ and ${\theta }_{{FC}}$ (FC stands for field capacity). Following ENVI-met standards, we will respectively call ${\theta }_{{Dry}}$ to a soil at the wilting point where $\theta =0 \%$; ${\theta }_{{ET}\max }$ to a soil that reached maximum ${E}_{T}$ and is at $\theta \,={\theta }_{{CRIT}}$, which was found to be $\theta =70 \%$ for the *Carpinus betulus* in Zurich’s soil conditions; and finally ${\theta }_{{Irr}}$ to a soil that is being irrigated and thus certainly is at least at a permanent state of field capacity, or $\theta =100 \%$. See supplementary materials, section S3, for the derivation of ${\theta }_{{CRIT}}$ and our data availability section for the detailed soil moisture content input in ENVI-met simulations. We consider that direct soil evaporation at wilting point is minimal, as water is tightly held by soil particles, hence we assume it is zero and we also assume interception evaporation as zero, given that days selected were summer days without rain.

Both WRF and ENVI-met use soil parameterisations anchored in locally derived soil thermal and hydraulic properties. Spatially distributed soil texture was sourced from local soil datasets and applied to both the WRF and ENVI-met control models to ensure consistency across scales. While WRF incorporates these properties in a more aggregated manner, the ENVI-met model employs a detailed representation of the soil characteristics. This approach guarantees that, despite differences in spatial resolution, the fundamental soil moisture dynamics are consistently represented in both models (see the data availability section for further details). ENVI-met allows for modifications in the engineered soil of the central square that can be used to create different scenarios of soil moisture content.

### Summer representative days

To understand the difference in tree cooling performance between most days in summer and the higher return period days of heat waves, we selected a representative historical average summer day (June 14th 2023) and a representative extreme day, or heat wave day (June 27th 2019). Average and extreme days were selected based on Zurich’s climatology and local weather stations. The heat wave day is defined by the Switzerland’s Federal Office of Meteorology and Climatology as the third consecutive day where average air temperatures were higher than 25 °C. Both average and extreme historical days were selected given the absence of precipitation and cloud cover, with preference to days as recent as possible and with the highest temperatures recorded. Then these days were used as input days in WRF.

### Vegetation

This research will focus on trees due to their superior cooling potential compared to grasses and shrubs due to the shading capacity of trees^19,26,63^. Additionally, to reduce the computational demand, we limited our scope to a single tree species, the Hornbeam (*Carpinus betulus*), the most common tree species in Zurich; this is a common approach seen in other studies^68^.

The ENVI-met 3D-Plant module explicitly creates a tree crown and root system by mesh generation and for each plant’s own specific shape^68^. In ENVI-met, the trees are not only modelled as porous media in relation to insolation and wind flow, but also interact with its surrounding environments by means of ${ET}$^65^. We varied the number of trees assessed from: a single tree (with a crown diameter of $9{m}$), to a small patch of six trees (total forested canopy diameter of $30{m}$), to a medium patch of 26 trees (total $48\,m$), up to a large patch of 74 trees (total $72\,m$). The final tree amounts in each patch do not follow a strict rule: we varied the size of the tree patch area by adding new trees in a pseudo-random way, mimicking how patches of trees are usually planted in Zurich (see supplementary materials, section S4, for more details). This approach generated a range of tree patch sizes and allowed us to analyse the sensitivity of cooling to different greenery scenarios while remaining within the constraints of the available empty space in the central square of the simplified neighbourhood models. The average distance between tree centroids is eight metres, with some density variations, providing adequate space for root development while representing a realistic planting layout.

The decision on simulating groups of trees together also results from the fact that trees have a synergistic cooling effect, meaning that the sum of cooling provided by an *n*-number of separate trees is not as good as the cooling provided by the same *n*-number of trees planted close together^78^. Ideally, we should aim for tree groups, as relying on single urban trees can increase ecological vulnerability, especially since street trees often grow under suboptimal conditions^79^. It is noteworthy that the adopted courtyard configuration of our ENVI-met model, with vegetation located in the centre of the courtyard, maximises the local cooling effect by optimising both shading and evapotranspiration. This setup represents a best-case scenario for tree cooling performance.

### Assessment of results

In our study, we used the UTCI index for thermal comfort, which has been widely adopted in outdoor thermal comfort studies^36^ and for its sensitivity to relative humidity. When comparing the UTCI from the no vegetation scenario (control, indicated by 'No veg.'), to the scenario with vegetation (indicated by 'Veg.'), under ${\theta }_{{Dry}}$ conditions, we expect to extract only the contribution of shading (Eq. 1):

1 $$\varDelta {{UTCI}}_{{Shade}}={\varDelta {UTCI}}^{{Dry}}$$

This is because we approximate the EC at permanent wilting point to zero. When comparing the $\varDelta {UTCI}$ from the control (no vegetation) scenario, to the scenarios with vegetation and high soil moisture conditions (${\theta }_{{ET}\max }$ and ${\theta }_{{Irr}}$), we can extract the combined effect of EC and shade. By removing the shade effect, we thereby isolated the EC effect (Eqs. 2, 3 and 4):

2 $${\varDelta {UTCI}}_{{EC}}^{{Dry}}=0$$

3 $${\varDelta {UTCI}}_{{EC}}^{{ET}\max }={\varDelta {UTCI}}^{{ET}\max }-{\varDelta {UTCI}}^{{Dry}}=\left({{UTCI}}_{{No\; veg}.}^{{ET}\max }-\,{{UTCI}}_{{Veg}.}^{{ET}\max }\right)-{\varDelta {UTCI}}^{{Dry}}$$

4 $${\varDelta {UTCI}}_{{EC}}^{{Irr}}={\varDelta {UTCI}}^{{Irr}}-{\varDelta {UTCI}}^{{Dry}}=\left({{UTCI}}_{{No\; veg}.}^{{Irr}}-\,{{UTCI}}_{{Veg}.}^{{Irr}}\right)-{\varDelta {UTCI}}^{{Dry}}$$

The thermal comfort impact of any vegetation addition in the centre of the models will be the combined impact of shade and EC (Eq. 5):

5 $$\varDelta {UTCI}=\varDelta {{UTCI}}_{{Shade}}+\varDelta {{UTCI}}_{{EC}}$$

For both ETmax and Irr scenarios, there is a cooling effect from canopy ET (${E}_{T}$) and direct soil evaporation (${E}_{S}$). Partitioning the EC effect of those is out of this study’s scope. We will focus on quantifying the total EC effect, which is found when comparing the Irr scenario to the Dry scenario (Eq. 6):

6 $$\varDelta {{UTCI}}_{{EC}}={\varDelta {UTCI}}^{{Irr}}-{\varDelta {UTCI}}^{{Dry}}$$

Our simulations show a significant decrease in cooling effects beyond the immediate tree canopy footprint. Therefore, we present results only from the model’s central point, excluding other areas. While shaded areas from surrounding buildings in the simplified neighbourhood model could also enhance pedestrian thermal comfort, they were not analysed.

### Data

All datasets used in the presented study and framework herein developed are listed in Table 1. In this table it is specified whether the datasets are local or global. For local datasets, the table also provides options for sourcing similar data globally available. Local datasets are preferred for their accuracy and up-to-dateness, though global datasets can also be sufficient.

## Supplementary information

Supplementary information

## Data Availability

The main input data used in WRF and ENVI-met models as well as the processed output data is publicly available at Eawag’s ERIC repository: 10.25678/000D4D.

## Code availability

The code used to turn our data into the figures currently published in this article is available at Eawag’s ERIC repository: 10.25678/000D4D. Code outputs were post-processed in page layout software to reach the graphical content of the figures displayed in this article.
