# Supplementary material for: Impact of soil moisture content on urban tree evaporative cooling and human thermal comfort
Source: NPJ Urban Sustain. 2025 May 16;5(1):26. doi: 10.1038/s42949-025-00220-0 (PMC12081291; doi:10.1038/s42949-025-00220-0)
Supplement: Supplementary file 1 — Supplementary information [file 42949_2025_220_MOESM1_ESM.pdf]

**Supplementary materials**

# Impact of soil moisture content on urban tree evaporative cooling and human thermal comfort

L. Gobatti[a,b]*, P. M. Bach[c,d], M. Maurer[a,b], J. P. Leitão[a,b]

[a] Swiss Federal Institute of Aquatic Science & Technology (Eawag), Überlandstrasse 133, Dübendorf, ZH 8600, Switzerland

[b] Institute of Environmental Engineering, ETH Zürich, Zürich, ZH 8093, Switzerland

[c] Department of Civil Engineering, 23 College Walk, Monash University Clayton 3800 VIC Australia

[d] EdenCT, Dübendorf, ZH 8600, Switzerland

*Corresponding author: lucas.gobatti@eawag.ch

## S1. Validation

The Index of Agreement (IoA) and Mean Absolute Error (MAE) results of the validation are presented in the manuscript just for the final ENVI-met + WRF models (the simplified neighbourhood models), and during the 9h-16h period. Figures S1 and S2 show the time series of air temperature and relative humidity, respectively, illustrating the limitations of the model. Tables S1 and S2 show the full error statistics, for both ENVI-met + WRF and WRF alone.

There is a correlation between the proximity to less dense Local Climate Zones (LCZs) and the model's ability to predict microclimate temperatures more effectively. These two observations are interconnected: limitations arise from the low resolution of mesoclimate-dependent weather data obtained from WRF, as well as the simplifications used to depict urban areas, which leads to the omission of certain urban thermal inertia effects that are better captured by microclimate models. Consequently, the heat island effects are dampened, and nighttime temperatures are inadequately described. As a result, our approach tends to slightly underestimate temperatures in denser LCZs and significantly underestimates nighttime heat.

Below are Eqs. S1, S2, S3, S4 and S5 for the minimum absolute error, maximum absolute error, Mean Absolute Error (MAE), Standard Deviation ($\sigma$) and Index of Agreement (IoA), respectively. Where $y_i$ represent the measured and $\hat{y}_i$ the modelled data at the $i$-th point, and $n$ is the number of data points.

$$Min.Abs.Error = min(|y_i - \hat{y}_i|) \tag{S1}$$

$$Max.Abs.Error = max(|y_i - \hat{y}_i|) \tag{S2}$$

$$MAE \ = \frac{1}{n}\sum_{i=1}^{n}|y_i - \hat{y}_i| \tag{S3}$$

$$\sigma \ = \sqrt{\frac{1}{n}\sum_{i=1}^{n}\left(y_i - \hat{y}_i - MAE\right)} \tag{S4}$$

$$IoA \ = 1 - \frac{\sum_{i=1}^{n}(y_i - \hat{y}_i)^2}{\sum_{i=1}^{n}(|\hat{y}_i - y_i| + |y_i - \hat{y}_i|)^2} \tag{S5}$$

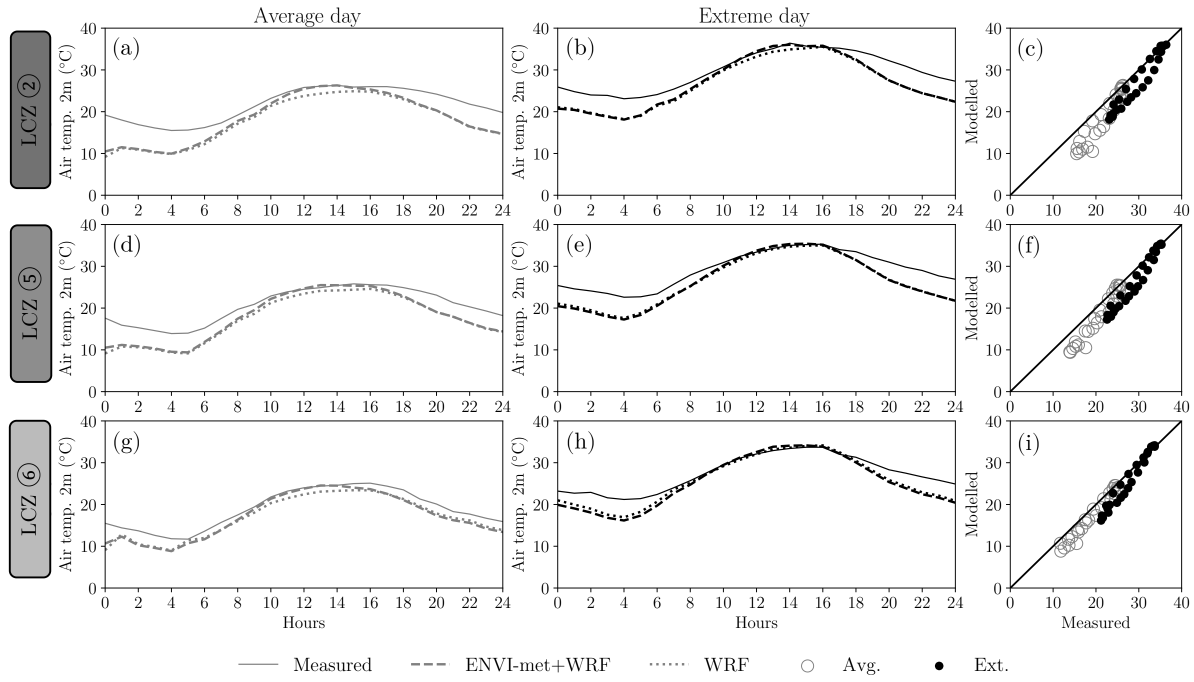


**Figure S1. Time series of air temperature and comparison between measured and modelled data.** Time Series of air temperature at 2.1m for each LCZ for the average and extreme summer days (a, b, d, e, g, h). Comparison between modelled and measured air temperatures in the centre of the simplified neighbourhood models in ENVI-met, using WRF LCZ-specific forcing, 2.1m above ground, for the Average and Extreme summer representative days (c, f, i).

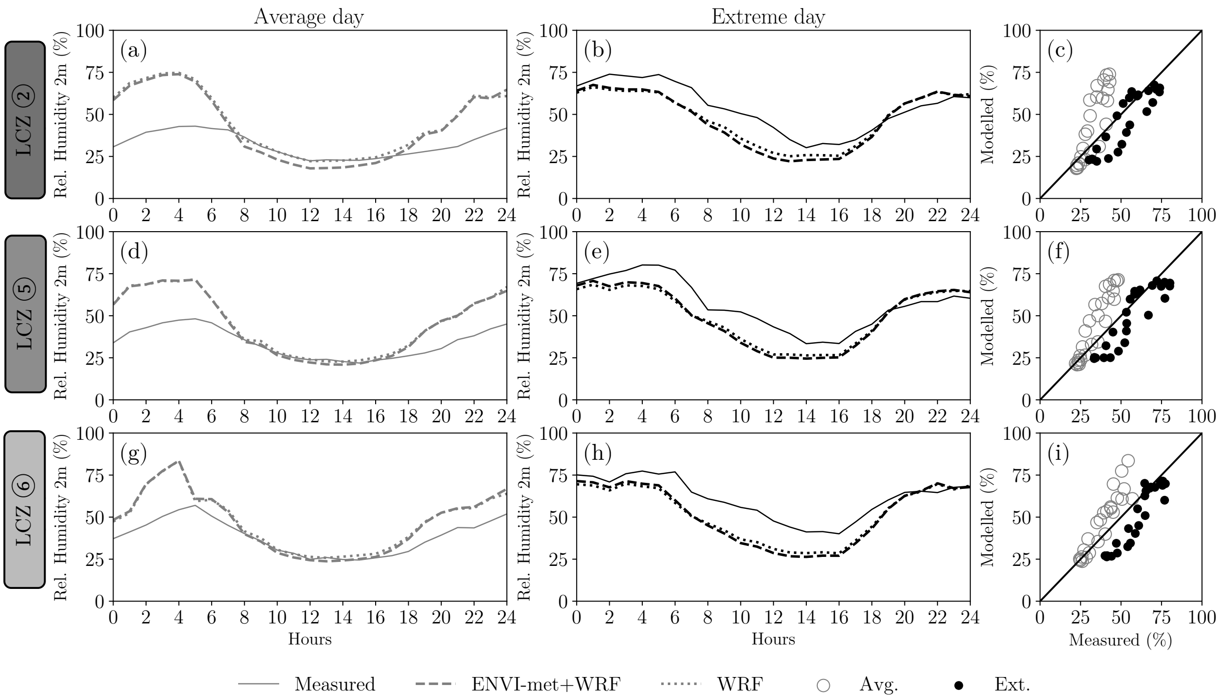


**Figure S2. Time series of relative humidity and comparison between measured and modelled data.** Time Series of relative humidity at 2.1m for each LCZ for the average and extreme summer days (a, b, d, e, g, h). Comparison between modelled and measured relative humidity in the centre of the simplified neighbourhood models in ENVI-met, using WRF LCZ-specific forcing, 2.1m above ground, for the Average and Extreme summer representative days (c, f, i).

| LCZ | Day | Model | Period | Min. Abs. Error | Mean Abs. Error | Max. Abs. Error | Standard Deviation | Index of Agreement |
|---|---|---|---|---|---|---|---|---|
| ② | Avg. | ENVI-met + WRF | 0-24 | 0.002 | 3.197 | 8.721 | 2.437 | 0.842 |
| | Avg. | ENVI-met + WRF | 9-16 | 0.002 | 0.618 | 1.894 | 0.663 | 0.954 |
| | Avg. | WRF | 0-24 | 1.008 | 3.773 | 10.014 | 2.177 | 0.812 |
| | Avg. | WRF | 9-16 | 1.008 | 1.718 | 2.491 | 0.468 | 0.823 |
| | Ext. | ENVI-met + WRF | 0-24 | 0.017 | 2.772 | 5.166 | 2.168 | 0.903 |
| | Ext. | ENVI-met + WRF | 9-16 | 0.017 | 0.435 | 1.052 | 0.569 | 0.990 |
| | Ext. | WRF | 0-24 | 0.144 | 2.950 | 5.071 | 1.813 | 0.897 |
| | Ext. | WRF | 9-16 | 0.144 | 0.864 | 1.502 | 0.509 | 0.967 |
| ⑤ | Avg. | ENVI-met + WRF | 0-24 | 0.069 | 2.702 | 7.081 | 1.987 | 0.896 |
| | Avg. | ENVI-met + WRF | 9-16 | 0.069 | 0.500 | 1.386 | 0.604 | 0.968 |
| | Avg. | WRF | 0-24 | 0.764 | 3.168 | 8.462 | 1.773 | 0.872 |
| | Avg. | WRF | 9-16 | 0.764 | 1.335 | 2.275 | 0.468 | 0.859 |
| | Ext. | ENVI-met + WRF | 0-24 | 0.127 | 2.877 | 5.551 | 2.188 | 0.894 |
| | Ext. | ENVI-met + WRF | 9-16 | 0.127 | 0.530 | 1.674 | 0.736 | 0.977 |
| | Ext. | WRF | 0-24 | 0.180 | 2.781 | 5.179 | 1.864 | 0.898 |
| | Ext. | WRF | 9-16 | 0.180 | 0.658 | 1.976 | 0.641 | 0.963 |
| ⑥ | Avg. | ENVI-met + WRF | 0-24 | 0.091 | 1.787 | 4.848 | 1.190 | 0.954 |
| | Avg. | ENVI-met + WRF | 9-16 | 0.091 | 0.526 | 1.426 | 0.502 | 0.965 |
| | Avg. | WRF | 0-24 | 0.260 | 1.958 | 6.436 | 1.098 | 0.945 |
| | Avg. | WRF | 9-16 | 1.324 | 1.511 | 1.713 | 0.161 | 0.856 |
| | Ext. | ENVI-met + WRF | 0-24 | 0.037 | 2.267 | 5.038 | 2.079 | 0.924 |
| | Ext. | ENVI-met + WRF | 9-16 | 0.037 | 0.390 | 0.920 | 0.399 | 0.988 |
| | Ext. | WRF | 0-24 | 0.084 | 1.813 | 4.262 | 1.699 | 0.944 |
| | Ext. | WRF | 9-16 | 0.084 | 0.297 | 0.446 | 0.338 | 0.995 |

**Table S1.** Error statistics for the comparison between modelled and measured air temperatures (shown in °C) in the centre of the simplified neighbourhood models in ENVI-met, using WRF LCZ-specific forcing, 2.1m above ground, for the Average and Extreme summer representative days.

| LCZ | Day | Model | Period | Min. Abs. Error | Mean Abs. Error | Max. Abs. Error | Standard Deviation | Index of Agreement |
|---|---|---|---|---|---|---|---|---|
| ② | Avg. | ENVI-met + WRF | 0-24 | 14.03 | 0.448 | 32.384 | 14.568 | 0.621 |
| | Avg. | ENVI-met + WRF | 9-16 | 4.045 | 2.605 | 4.902 | 0.861 | 0.68 |
| | Avg. | WRF | 0-24 | 13.342 | 0.004 | 33.463 | 13.164 | 0.595 |
| | Avg. | WRF | 9-16 | 0.488 | 0.004 | 1.167 | 0.628 | 0.99 |
| | Ext. | ENVI-met + WRF | 0-24 | 8.701 | 1.025 | 20.529 | 7.592 | 0.891 |
| | Ext. | ENVI-met + WRF | 9-16 | 13.696 | 7.302 | 20.529 | 4.999 | 0.573 |
| | Ext. | WRF | 0-24 | 7.787 | 0.8 | 17 | 6.734 | 0.907 |
| | Ext. | WRF | 9-16 | 10.725 | 4.4 | 17 | 4.579 | 0.654 |
| ⑤ | Avg. | ENVI-met + WRF | 0-24 | 11.502 | 0.062 | 27.108 | 10.873 | 0.735 |
| | Avg. | ENVI-met + WRF | 9-16 | 1.319 | 0.062 | 2.981 | 1.264 | 0.947 |
| | Avg. | WRF | 0-24 | 11.593 | 0.067 | 27.566 | 10.2 | 0.725 |
| | Avg. | WRF | 9-16 | 1.107 | 0.067 | 3.181 | 1.322 | 0.954 |
| | Ext. | ENVI-met + WRF | 0-24 | 9.084 | 1.188 | 19.44 | 7.892 | 0.89 |
| | Ext. | ENVI-met + WRF | 9-16 | 13.656 | 8.252 | 19.44 | 4.635 | 0.545 |
| | Ext. | WRF | 0-24 | 8.834 | 1.1 | 18.2 | 7.433 | 0.89 |
| | Ext. | WRF | 9-16 | 11.762 | 7 | 17.3 | 4.411 | 0.593 |
| ⑥ | Avg. | ENVI-met + WRF | 0-24 | 8.93 | 0.294 | 29.134 | 9.133 | 0.82 |
| | Avg. | ENVI-met + WRF | 9-16 | 1.109 | 0.319 | 2.485 | 1.173 | 0.964 |
| | Avg. | WRF | 0-24 | 8.912 | 0.2 | 29.1 | 8.263 | 0.815 |
| | Avg. | WRF | 9-16 | 1.388 | 0.2 | 2.8 | 1.228 | 0.948 |
| | Ext. | ENVI-met + WRF | 0-24 | 10.157 | 0.207 | 21.655 | 7.669 | 0.838 |
| | Ext. | ENVI-met + WRF | 9-16 | 17.502 | 13.051 | 21.655 | 3.204 | 0.455 |
| | Ext. | WRF | 0-24 | 9.79 | 0.2 | 19 | 6.686 | 0.846 |
| | Ext. | WRF | 9-16 | 15.35 | 11.6 | 19 | 2.946 | 0.496 |

**Table S2.** Error statistics for the comparison between modelled and measured relative humidity (shown in %) in the centre of the simplified neighbourhood models in ENVI-met, using WRF LCZ-specific forcing, 2.1m above ground, for the Average and Extreme summer representative days.

## S2. Logarithmic fit

Table S3 indicates the statistics of the natural logarithmic fit used in Figure 8 of the paper.

| LCZ | Day | SMC | IoA | Min. Abs. Error | Mean Abs. Error | Max. Abs. Error | Standard Deviation |
|---|---|---|---|---|---|---|---|
| ② | Avg. | Dry (0%) | 0.999715 | 0.008509 | 0.023548 | 0.038586 | 0.014907 |
| | Avg. | ETmax (70%) | 0.999289 | 0.012099 | 0.040957 | 0.081914 | 0.026195 |
| | Avg. | Irr (100%) | 0.995562 | 0.069607 | 0.159213 | 0.318426 | 0.095471 |
| | Ext. | Dry (0%) | 0.998328 | 0.027279 | 0.057016 | 0.114033 | 0.033736 |
| | Ext. | ETmax (70%) | 0.997761 | 0.032708 | 0.069910 | 0.139819 | 0.041496 |
| | Ext. | Irr (100%) | 0.993869 | 0.088824 | 0.178469 | 0.356938 | 0.105043 |
| ⑤ | Avg. | Dry (0%) | 0.998306 | 0.033647 | 0.053966 | 0.074286 | 0.014423 |
| | Avg. | ETmax (70%) | 0.999176 | 0.017441 | 0.038304 | 0.059167 | 0.020157 |
| | Avg. | Irr (100%) | 0.996345 | 0.042581 | 0.131206 | 0.219832 | 0.084327 |
| | Ext. | Dry (0%) | 0.998138 | 0.034368 | 0.057445 | 0.114889 | 0.033254 |
| | Ext. | ETmax (70%) | 0.997376 | 0.046977 | 0.073282 | 0.146565 | 0.042369 |
| | Ext. | Irr (100%) | 0.993698 | 0.059449 | 0.175622 | 0.351244 | 0.109850 |
| ⑥ | Avg. | Dry (0%) | 0.999246 | 0.024003 | 0.037582 | 0.051161 | 0.009965 |
| | Avg. | ETmax (70%) | 0.999723 | 0.002544 | 0.020920 | 0.039295 | 0.015591 |
| | Avg. | Irr (100%) | 0.996711 | 0.003486 | 0.120114 | 0.236741 | 0.091024 |
| | Ext. | Dry (0%) | 0.997706 | 0.024113 | 0.065017 | 0.130034 | 0.039396 |
| | Ext. | ETmax (70%) | 0.996776 | 0.027133 | 0.083053 | 0.166106 | 0.051088 |
| | Ext. | Irr (100%) | 0.993356 | 0.098519 | 0.188038 | 0.376075 | 0.109998 |

**Table S3.** Error statistics for the comparison between fitted and modelled progress of thermal comfort as greenery increases relative to the control scenario without trees, at 2.1m above ground in the centre of the model domain at 14h.

## S3. Estimating S*

In the manuscript, we show that an $\theta_{CRIT}$ is the point of incipient stomatal closure, which is related to the vegetation type and soil type. In this section we dive into details for quantifying this point.

The total control volume of a soil parcel $V_s$ can be expressed as a combination of (Rodríguez-Iturbe and Porporato, 2005):

$$V_s = V_A + V_W + V_m \tag{S6}$$

Where $V_A$ is the dynamic volume of air, $V_W$ the dynamic volume of water and $V_m$ is the (here considered constant, but in longer time scales is also dynamic) volume of minerals. Thus, the porosity $n$ of the soil is also constant and defined as (Rodríguez-Iturbe and Porporato, 2005):

$$n = \frac{V_A + V_W}{V_s} \tag{S7}$$

Meaning that for every water parcel entering the system, mass will be conserved while an air parcel equal in volume will leave the system. The water within a soil can be divided into (Gobatti and Leite, 2023):

$$max(V_W) = V_{hW} + V_{cW} + V_{gW} \tag{S8}$$

Where $V_{hW}$ is the hygroscopic water component, firmly adhered to soil particles by surface tension, not available to plants; $V_{cW}$ is the capillary water component, or the Plant Available Water (PAW), present in the soil pores and around soil particles, maintained in the soil by adhesion and cohesion; and $V_{gW}$, the gravitational water, is held loosely in the soil, as it cannot be contained by adhesion or cohesion forces, being unavailable to plants and easily dragged by gravity and creating leakage and runoff (O'Geen, 2013).

In ENVI-met version 5.6, the relative soil moisture $\theta$, function of $V_W$, varies from Oven Dry (OD) to Field Capacity (FC) conditions. This means it goes from where there is theoretically no water in the system (OD), all the way to where the system is maximised on hygroscopic and capillary water (FC), about to reach gravitational water (see Figure S3).

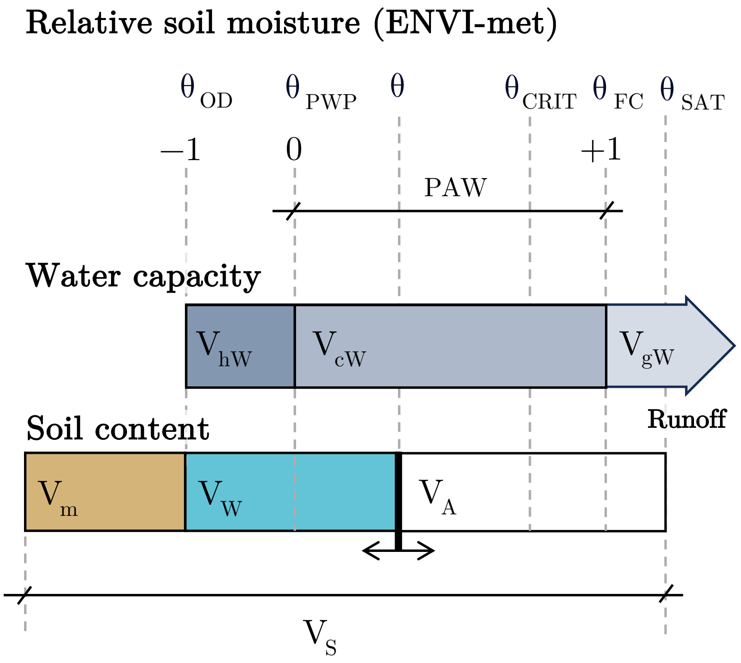


**Figure S3.** ENVI-met Version 5.6 relative soil moisture content scheme. $S$ is the relative soil moisture content, and $\theta_{OD}$, $\theta_{PWP}$, $\theta_{CRIT}$, $\theta_{FC}$, $\theta_{SAT}$ are respectively the relative soil moisture content at Oven Dry ($\theta = -1$), Permanent Wilting Point ($\theta = 0$), point of incipient stomatal closure ($\theta_{CRIT}$), Field Capacity ($\theta = 1$) and saturated ($\theta > 1$) conditions. The volumes $V_{hW}$ represent the hygroscopic water volume, $V_{cW}$ the capillary water volume, $V_{gW}$ the gravitational water volume, $\underline{V_m}$ the volume of minerals in a soil parcel, $V_W$ the dynamic water volume in a soil parcel, $V_A$ the dynamic air volume in a soil parcel, $\underline{V_s}$ the total soil volume. PAW signals the Plant Available Water region.

In ENVI-met, when $0 \leq \theta \leq 1$ it represents the fraction of the Plant Available Water that is actually available in the system; and when $-1 \leq \theta < \mathbf{0}$, it represents the fraction of hygroscopic water available in the system:

$$\theta(V_W) = \frac{V_W - V_{hW}}{V_{cW}}, V_W \in [V_{hW}, V_{hW} + V_{cW}], 0 \leq \theta \leq 1$$
$$\theta(V_W) = \frac{V_W}{V_{hW}} - 1, V_W \in [0, V_{hW}], -1 \leq \theta < 0 \qquad \text{(S9)}$$

In practice, plant transpiration happens mostly within the Plant Available Water region (Rodríguez-Iturbe and Porporato, 2005). For that reason, this study will focus on characterising evaporative cooling for $S$ ranging from the Permanent Wilting Point ($\theta \rightarrow 0$) to Field Capacity ($\theta \rightarrow 1$).

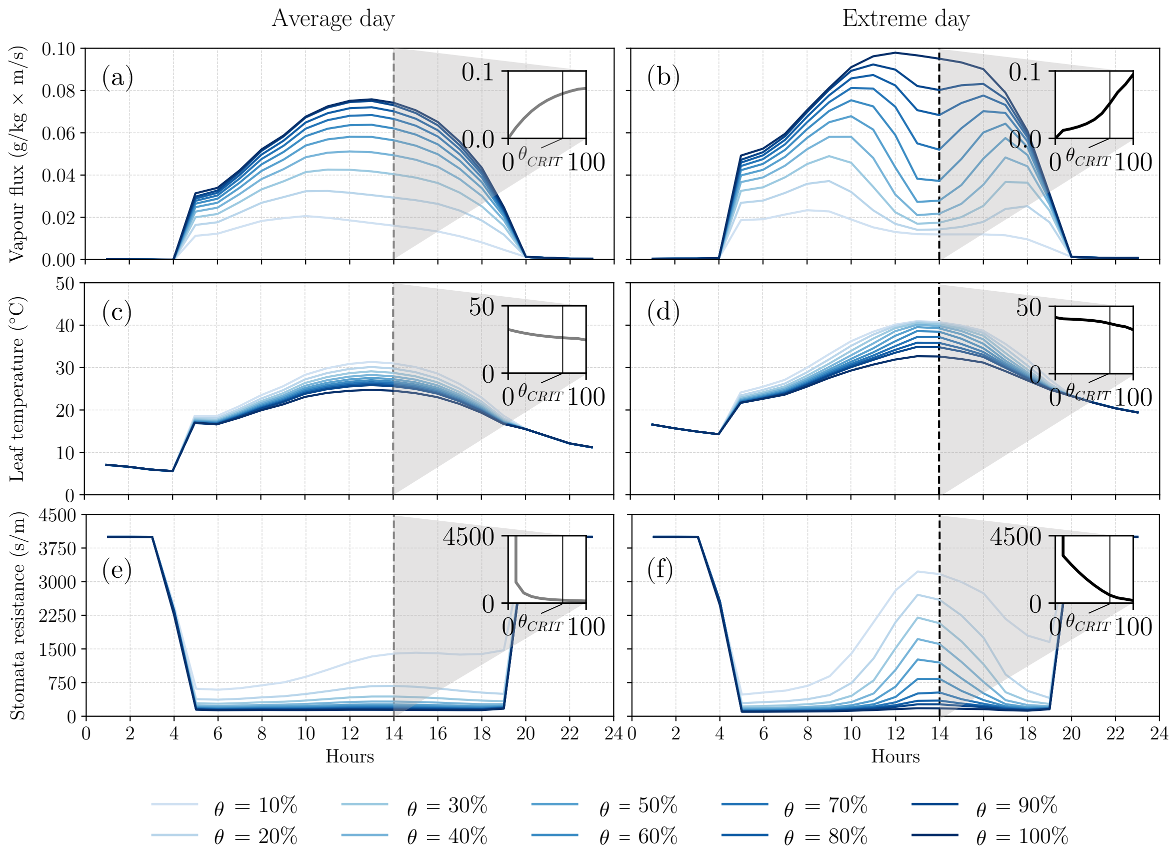


**Figure S4. Vapour flux (a, b), leaf temperature (c, d) and stomata resistance (e, f), for the average and extreme summer representative days.** The lines represent the different relative soil moisture contents. Values for the permanent wilting point (PWP) are not graphically represented because stomata resistance simply tends to be infinite. The scenario where $\theta$ = 1 also has irrigation added, meaning that the soil is always at field capacity (FC). The small plots within each subplot show results for 14h in different soil moisture contents.

The single *Carpinus betulus* tree simulation results for the different summer representative days and different relative soil moisture contents are represented in Figure S4. Vapour flux will increase when soil moisture content increases, given there is an increase in evapotranspiration ET. Evaporative cooling increases as soil moisture content increases. The stomata resistance also increases during higher temperatures.

However, the most relevant use of Figure S4 is to find the $\theta_{CRIT}$. Finding the moment when temperature is the highest during the average and extreme summer day scenarios, as well as the $ET$ behaviour of the *Carpinus betulus* at that time frame are the means to reach $\theta_{CRIT}$. The higher temperatures were found to be at 14h for both

scenarios, and a smaller plot indicates the dynamic of each relevant phenomena given differences in relative soil moisture content. As stomatal resistance is an indicator of $ET$, as well as vapour flux, from their behaviour we learned that the $\theta_{CRIT}$ can be different given different surrounding heat conditions, that are expressed in the leaf temperature.

This ratio between the latent heat flux and the total heat flux over a surface is denoted by evaporative fraction (Seneviratne et al., 2010). This fraction has a maximum value ($EF_{max}$) which can be soil moisture limited or energy limited (Seneviratne et al., 2010). In our case, it means when there is not enough water in the soil to sustain transpiration or not enough heat to evaporate this transpiration, actual $ET$ is smaller than $ET_{max}$. This phenomenon can be observed in the 14h results for vapour flux: up to a threshold around $\theta_{CRIT}$ of relative soil moisture, the vapour flux established during the average day, likely energy limited. However, during the extreme day, as S increased, vapour flux from $ET$ kept increasing, as it was likely not heat limited. On the other hand, although $ET$ was less energy limited during the extreme day, $ET$ was in general higher in the average day for $\theta < \theta_{CRIT}$. This result makes sense, given that the stomata resistance was also higher through most of the extreme day in comparison to the average day.

The combination of these results leads us to believe that the $\theta_{CRIT}$, represents the point of incipient stomatal closure indicated by Rodríguez-Iturbe and Porporato (2005). This point was found by analysing the stomatal resistance, which is a phenomenon that covers the entire scale of water stress (Hsiao, 1973; Porporato et al., 2001). We estimated graphically when stomata start to close during the less heat restricted scenario (14h of the extreme summer day), meaning that soil moisture content is getting below the $\theta_{CRIT}$, finding that $\theta_{CRIT} = 0.7$. This value corresponds only to the *Carpinus betulus* species, which is the only species that will be used in this study, as well as the local soil used in ENVI-met, also the only soil used for all archetypes.

## S4. Simplified Neighbourhood Models in ENVI-met

Below in Figure S5 you may find the images of the 2D versions of the .INX models in ENVI-met for all three LCZs. In Figure S6 you can see the different tree patches variations for LCZ 2.

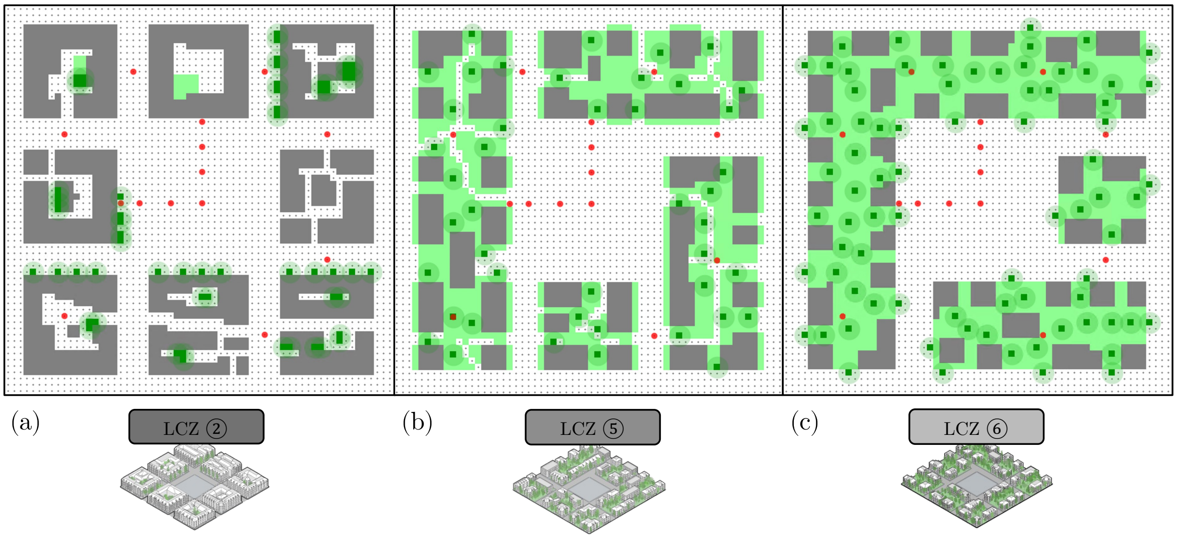


**Figure S5. Local Climate Zones 2 (a), 5 (b) and 6 (c) and their respective .INX models in ENVI-met.** Grey colours represent buildings, light green represent grass, dark green and a buffer represent Hornbeam trees and red are receptors.

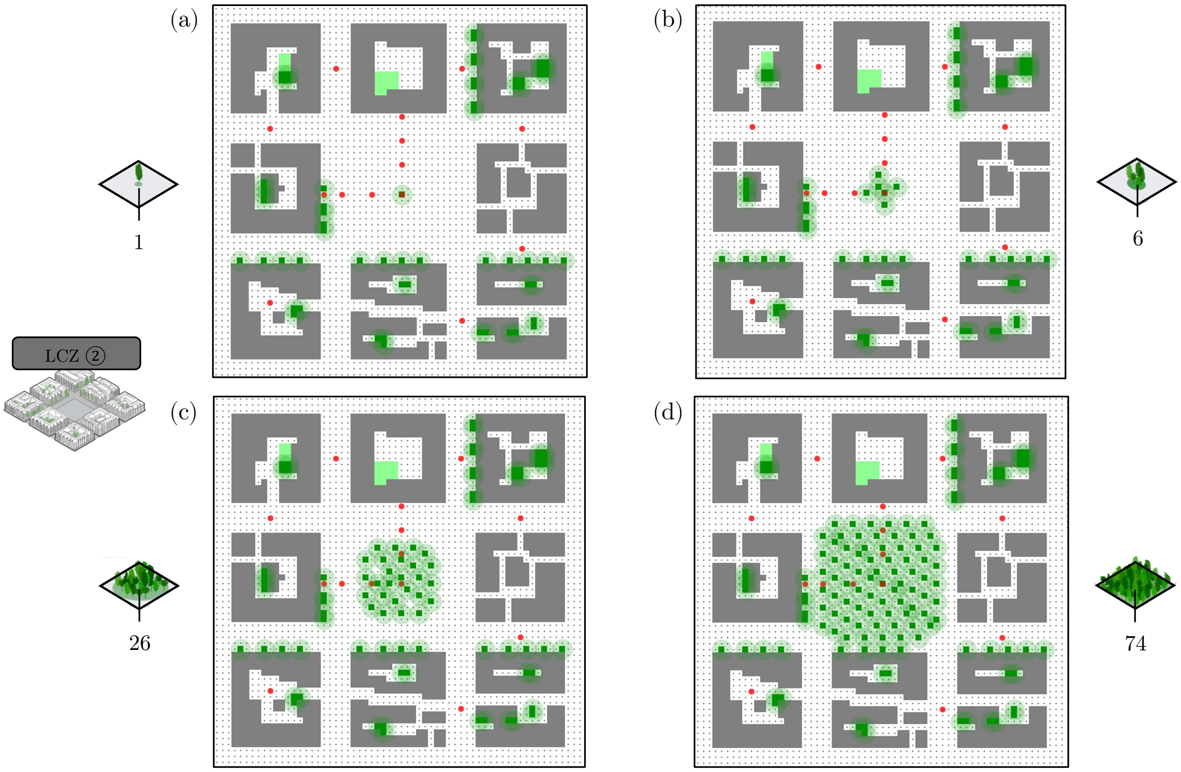


**Figure S6. Local Climate Zone 2 and their respective .INX models in ENVI-met for different tree amounts.** Single tree (a), small patch of trees (b), medium patch of trees (c) and large patch of trees (d). Grey colours represent buildings, light green represent grass, dark green and a buffer represent Hornbeam trees and red are receptors.

## REFERENCES


Gobatti, L., Leite, B.C.C., 2023. Unmanaged vegetated roofs hydrological performance in subtropical areas: An investigation in São Paulo, Brazil. Sci. Total Environ. 874, 162417. https://doi.org/10.1016/j.scitotenv.2023.162417

Rodríguez-Iturbe, I., Porporato, A., 2005. Ecohydrology of Water-Controlled Ecosystems: Soil Moisture and Plant Dynamics, 1st ed. Cambridge University Press. https://doi.org/10.1017/CBO9780511535727

Seneviratne, S.I., Corti, T., Davin, E.L., Hirschi, M., Jaeger, E.B., Lehner, I., Orlowsky, B., Teuling, A.J., 2010. Investigating soil moisture–climate interactions in a changing climate: A review. Earth-Sci. Rev. 99, 125–161. https://doi.org/10.1016/j.earscirev.2010.02.004
